# Mechanistic insights into synergy between nalidixic acid and tetracycline against clinical isolates of *Acinetobacter baumannii* and *Escherichia coli*

**DOI:** 10.1038/s42003-021-02074-5

**Published:** 2021-05-10

**Authors:** Amit Gaurav, Varsha Gupta, Sandeep K. Shrivastava, Ranjana Pathania

**Affiliations:** 1 Department of Biotechnology, Indian Institute of Technology Roorkee, Roorkee, Uttarakhand India; 2 Department of Microbiology, Government Medical College and Hospital Chandigarh, Chandigarh, Punjab India; 3 Centre for Innovation, Research and Development, Dr. B. Lal Clinical Laboratory Pvt Ltd, Jaipur, Rajasthan India

**Keywords:** Antimicrobials, Clinical microbiology, Microbiology

## Abstract

The increasing prevalence of antimicrobial resistance has become a global health problem. *Acinetobacter baumannii* is an important nosocomial pathogen due to its capacity to persist in the hospital environment. It has a high mortality rate and few treatment options. Antibiotic combinations can help to fight multi-drug resistant (MDR) bacterial infections, but they are rarely used in the clinics and mostly unexplored. The interaction between bacteriostatic and bactericidal antibiotics are mostly reported as antagonism based on the results obtained in the susceptible model laboratory strain *Escherichia coli*. However, in the present study, we report a synergistic interaction between nalidixic acid and tetracycline against clinical multi-drug resistant *A. baumannii* and *E. coli*. Here we provide mechanistic insight into this dichotomy. The synergistic combination was studied by checkerboard assay and time-kill curve analysis. We also elucidate the mechanism behind this synergy using several techniques such as fluorescence spectroscopy, flow cytometry, fluorescence microscopy, morphometric analysis, and real-time polymerase chain reaction. Nalidixic acid and tetracycline combination displayed synergy against most of the MDR clinical isolates of *A. baumannii* and *E. coli* but not against susceptible isolates. Finally, we demonstrate that this combination is also effective in vivo in an *A. baumannii*/*Caenorhabditis elegans* infection model (*p* < 0.001)

## Abstract (web summary)

Gaurav et al. report a synergistic interaction between nalidixic acid and tetracycline against multi-drug resistant bacteria *Acinetobacter baumannii* and *Escherichia coli*. They find that this synergy does not work for clinical isolates susceptible to antibiotics, suggesting the potential application of nalidixic acid and tetracycline against the emergence of multi-drug resistant bacteria.

## Introduction

The spread of antibiotic resistance is a serious threat to modern medicine. The continuing rise of antibiotic resistance in nosocomial pathogens is a grave public health concern. *Acinetobacter baumannii* is one of the major causes of hospital-acquired infections worldwide. The World Health Organization (WHO) has declared it as one of the most serious pathogens and is included in priority 1: “critical group” in the global priority pathogens list^1^. It is estimated that *Acinetobacter* spp. infects 600,000 to 1,400,000 people globally per year^2^. The remarkable ability of this pathogen to acquire resistance to almost all currently used antibiotics, including carbapenems, makes the situation even worse. The extreme drug resistance phenotype of *A. baumannii* shows a mortality rate of 70% in some cases^2^. The treatment options available against this pathogen are shrinking faster than the discovery rate of new antibacterials. Combining antibiotics is a promising strategy for increasing treatment efficacy and for controlling resistance evolution^3,4^. Despite their growing biomedical importance, fundamental questions about drug interactions remain unanswered. Especially, little is known about the underlying mechanisms of most antibiotic interactions^5,6^. Characterization of the underlying mechanisms of antibiotic interactions is important. The fundamental antibiotics’ modes of action and pharmacodynamics alone cannot elucidate drug interactions in a simple, straightforward way. Drug interactions can be caused by relatively simple uptake effects; e.g., synergism results if one drug increases the permeability of the cell envelope to another drug^4,7,8^.

Antibiotic combinations have several benefits such as broadening the antibiotic spectrum and reduced toxicity, but slowing the evolution of drug resistance is a key motivation for using drug combinations^9^. Typically, several independent mutations are required to become resistant to a combination of drugs with different cellular targets^5^. Identification of drug interactions is important, because drugs may interact differently in different resistant mutants, i.e., acquired drug resistance affects the interactions between two antibiotics observed initially in wild-type drug-sensitive cells. Most of the existing knowledge about antibiotic interactions only represents data from large screening sets conducted in *Escherichia coli* (model laboratory strain) rather than multidrug-resistant (MDR) isolates. Further, the results point out towards antagonism between bacteriostatic and bactericidal antibiotics^10–12^. Few studies on drug interaction have been conducted in MDR isolates that showed positive drug interaction, i.e., synergism between bacteriostatic and bactericidal antibiotics^13,14^.

However, here we present a study describing the identification of a synergistic combination of nalidixic acid and tetracycline against MDR *A. baumannii* AYE (an epidemic strain in France). We extended our investigation of this synergistic combination against clinical isolates of *A. baumannii* and other human pathogens, including MDR *E. coli* (a WHO priority pathogen), and finally demonstrated the in vivo efficacy of this synergy using *Caenorhabditis elegans* infection model; our results highlight the dichotomy about antibiotic interactions. In addition, the underlying mechanism of this antibiotic interaction was explored using several different experimental approaches. The previous reports of antagonism between DNA synthesis inhibitors and translation inhibitors in *E. coli* do not seem to be universal for all other species.

## Results

### Screening of antibiotic–antibiotic combinations identify nalidixic acid and tetracycline synergism against *A. baumannii* AYE

**Fig. 1:**
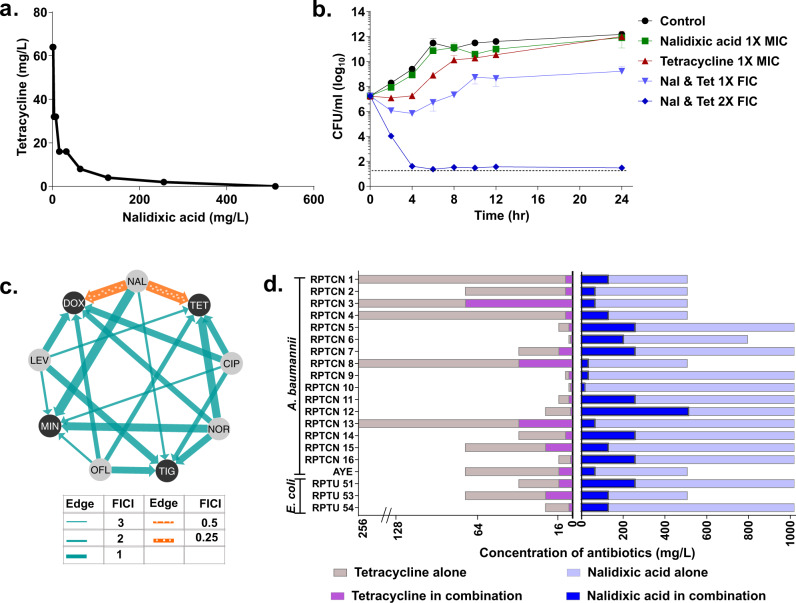
Nalidixic acid and tetracycline synergy against *A. baumannii* and *E. coli*. **a** Isobologram of nalidixic acid and tetracycline showing synergistic action of two antibiotics against *A. baumannii* AYE. **b** Time-kill curve of nalidixic acid and tetracycline combination against *A. baumannii* AYE: nalidixic acid and tetracycline at 1× FIC (0.25× MIC of both antibiotics) decreased the bacterial count by 2 log_10_ CFU/ml, whereas 2× FIC (0.5× MIC of both antibiotics) decreased bacterial count by 6 log_10_ CFU/ml. The CFU detection limit in this experiment corresponds to 30 CFU/mL. Data are represented as mean with SD from three independent experiments. **c** Drug–drug interaction network of quinolone and tetracycline class in *A. baumannii* AYE. Nodes represent different antibiotics, edges represent synergy (orange) or no interaction (blue), and thickness reflects the Fractional Inhibitory Concentration Index (FICI). Interaction network was created with Cytoscape version 3.8.0. CIP, ciprofloxacin; DOX, doxycycline; LEV, levofloxacin; MIN, minocycline; NAL, nalidixic acid; NOR, norfloxacin; OFL, ofloxacin; TET, tetracycline; TIG, tigecycline. **d** Concentration of tetracycline (dark purple) and nalidixic acid (dark blue) in combination, compared to tetracycline alone (light pink) and nalidixic acid alone (light blue) in clinical strains of *A. baumannii* and *E. coli*.

In this screening, two antibiotics, nalidixic acid and ciprofloxacin (Quinolones), were probed against antibiotics from different classes representing major classes of antibiotics such as ampicillin (β-lactams), fosfomycin (Phosphonates), gentamicin (Aminoglycosides), erythromycin (Macrolides), polymyxin B (Polymyxins), tetracycline (Tetracyclines), and rifampicin (Ansamycins) against MDR *A. baumannii* AYE (an epidemic strain in France)^15^. Antibiotic interaction was studied using a two-dimensional checkerboard assay to identify the synergistic combinations. This screening identified one potent combination, i.e., nalidixic acid and tetracycline showing a Fractional Inhibitory Concentration Index (FICI) value of 0.25. Isobologram showed a synergistic growth inhibition pattern of *A. baumannii* AYE (Fig. 1a). Most of the antibiotic–antibiotic interactions displayed no interaction. The results of this screening are shown in Supplementary Fig. 1 and Supplementary Tables 1 and 2. Next, we performed a time-kill curve of *A. baumannii* AYE exposed to nalidixic acid (at 512 mg/L), tetracycline (at 64 mg/L) alone or in combination (1× fractional inhibitory concentration (FIC) represents nalidixic acid and tetracycline at 64 and 8 mg/L, respectively; 2× FIC represents nalidixic acid and tetracycline at 128 and 16 mg/L, respectively). The time-kill kinetics also showed a synergistic pattern of killing at 1× FIC. This combination showed a colony forming unit (CFU) log reduction up to 2 log_10_ and 6 log_10_ at 1× FIC and 2× FIC, respectively, within 6 h (Fig. 1b). Nalidixic acid and tetracycline alone, even at higher concentrations, i.e., 1× minimum inhibitory concentration (MIC), were not able to decrease CFU counts significantly. Next, we also tested antibiotic interactions between other members of the quinolone and tetracycline classes. We selected nalidixic acid, ofloxacin, levofloxacin, and norfloxacin from the quinolone class and tetracycline, doxycycline, minocycline, and tigecycline from the tetracycline class. Most of these interactions displayed no interaction, except nalidixic acid and doxycycline, which displayed synergy with an FICI of 0.256 (Fig. 1c).

### The synergistic combination of nalidixic acid and tetracycline also works against MDR clinical strains of *A. baumannii* and *E. coli*

Next, we tested the synergistic combination of nalidixic acid and tetracycline against 16 MDR clinical strains of *A. baumannii* (all strains resistant to last-resort antibiotics including meropenem, amikacin, gentamicin, ciprofloxacin, and Co-trimoxazole). We also tested this combination against a panel of WHO priority pathogens such as *E. coli*, *Staphylococcus aureus*, *Klebsiella pneumoniae*, *Pseudomonas aeruginosa*, and some other human pathogens such as *Mycobacterium smegmatis*, *Shigella flexneri*, and *Salmonella enterica* subsp*. enterica* serovar Choleraesuis. The FICI data of these clinical strains are summarized in Supplementary Table 3. This synergy worked not only in the reference strain (i.e., *A. baumannii* AYE) but also against MDR clinical isolates of *A. baumannii* and *E. coli* (Fig. 1d). The combination works synergistically in quinolone-resistant clinical strains of *A. baumannii* (*n* = 16) (FICI range 0.1875–0.5 for 12 and 0.5–0.75 for 4 strains) and *E. coli* (*n* = 3) (FICI range 0.25–0.5) but not in quinolone-sensitive *E. coli* MG1655. When we tested the synergistic combination of nalidixic acid and doxycycline against clinical strains of *A. baumannii* and *E. coli*, it showed synergy against only six clinical strains and, hence, we did not proceed further with this combination (results are summarized in Supplementary Fig. 2 and Supplementary Table 4).

### Identification of the mechanism of synergy between nalidixic acid and tetracycline: nalidixic acid facilitates the entry of tetracycline inside *A. baumannii* AYE cells

**Fig. 2:**
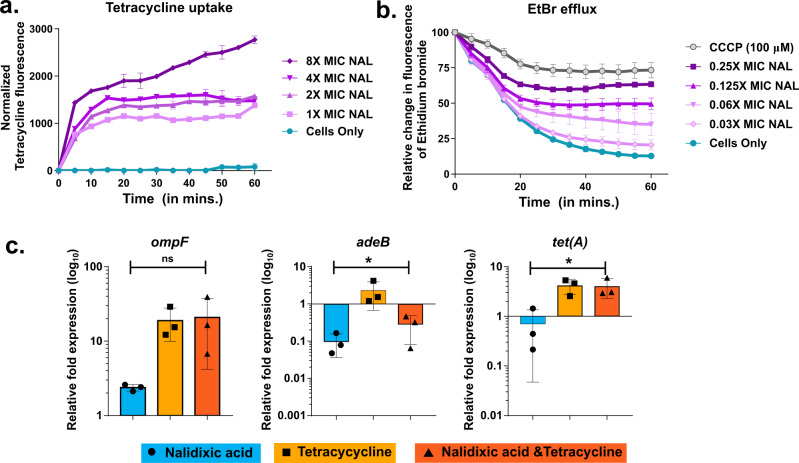
Role of nalidixic acid in tetracycline entry into cell. **a** Tetracycline uptake assay. Nalidixic acid enhances the uptake of tetracycline in *A. baumannii* AYE. The concentration of tetracycline was kept constant at 128 mg/L, which is not inhibitory for OD_600nm_ ~ 0.5 cells. The increasing concentration of nalidixic acid was added (here, 1× MIC corresponds to 512 mg/L), as indicated in the subset above. Control cells represent bacterial cells without nalidixic acid treatment but with the same amount of tetracycline. Tetracycline does not show intrinsic fluorescence as “cells only,” i.e., cells without nalidixic acid treatment do not show an increase in fluorescence with time. Data are normalized with respect to tetracycline fluorescence at time zero. Data are represented as mean with SD from two independent experiments. **b** Ethidium bromide (EtBr) efflux inhibition assay. Increasing concentration of nalidixic acid caused a concentration-dependent decrease in efflux of EtBr (a common substrate for many efflux pumps). *A. baumannii* AYE cells without nalidixic acid “Cells only” caused maximum efflux of EtBr. Proton uncoupler carbonyl cyanide m-chlorophenyl hydrazone (CCCP) acts as efflux pump inhibitor (positive control) for this experiment. **c** Relative expression of tetracycline importer, i.e., outer membrane protein (*omp33*) and tetracycline exporters (efflux pump) *adeB* and *tet(A)* after nalidixic acid, tetracycline, or a combination of both (at 0.75× MIC). Expression was measured by quantitative RT-PCR and normalized to no-drug treatment in *A. baumannii* AYE. Data represent three independent biological replicates.

The synergy between two compounds can be due to enhanced uptake of one by another partner; synergy on the basis of enhanced uptake has been proven in earlier studies^8,16^. Tetracycline is a fluorescent molecule and the fluorescence of tetracycline is enhanced when it enters inside the bacterial cells. To elucidate the mechanism of synergy of nalidixic acid and tetracycline, tetracycline uptake assay was performed. Here, nalidixic acid caused a concentration-dependent increase (ranging from 1× MIC to 8× MIC) in the uptake of tetracycline (as evident by an increase in fluorescence) (Fig. 2a), indicating that nalidixic acid helps tetracycline to get inside the *A. baumannii* AYE cells. In a similar set of experiments, tetracycline uptake assay was performed with clinical strain of *E. coli* (*E. coli* RPTU54, an MDR strain), *A. baumannii* SDF, and *E. coli* MG1655 (both strains are susceptible to nalidixic acid). An increasing concentration of nalidixic acid (ranging from 1× MIC to 8× MIC) did not change the relative fluorescence of tetracycline in the susceptible strains *A. baumannii* SDF and *E. coli* MG1655, whereas it showed enhanced uptake in MDR *E. coli* RPTU54 (Supplementary Fig. 3), indicating that nalidixic acid (at lower concentration) does not enhance uptake of tetracycline in susceptible bacteria. We also tested whether other quinolones such as ciprofloxacin can also enhance tetracycline uptake. We found that increasing concentration of ciprofloxacin did not result in enhanced fluorescence of tetracycline (Supplementary Fig. 4). This may explain why nalidixic acid displayed synergy with tetracycline, while ciprofloxacin did not.

Next, we tested whether the presence of nalidixic acid is affecting tetracycline transporters in *A. baumannii* AYE. Tetracycline gets inside the bacterial cell via outer membrane proteins (OMPs). Omp33 is a major OMP in *A. baumannii* AYE. Hence, we tested the expression level of *omp33* after nalidixic acid treatment. We found that nalidixic acid enhanced the relative expression of *omp33* up to twofold in *A. baumannii* AYE (Fig. 2c). Similarly, Many MDR strains such as *A. baumannii* AYE efflux out tetracycline by efflux pumps. Hence, we also tested the effect of nalidixic acid on the expression of two important efflux pumps that are responsible for tetracycline efflux, i.e., AdeB and Tet(A). We found that nalidixic acid caused a downregulation in the expression of both efflux pumps, i.e., *adeB* and *tet(A)* by ~21 and ~2-fold, respectively, (Fig. 2c). Enhanced uptake and reduced efflux of tetracycline by nalidixic acid may partially explain the basis of synergy between these two partners.

### Assessment of membrane damage in *A. baumannii* AYE

**Fig. 3:**
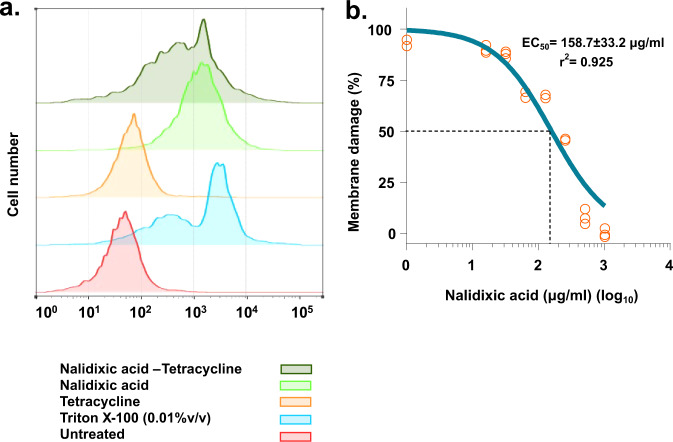
Role of nalidixic acid in membrane damage. **a** Flow cytometry analysis (Half Offset graphs) showing the role of nalidixic acid in membrane damage. Membrane damage was assessed using membrane-impermeable dye SYTOX^TM^ Orange. Nalidixic acid treatment causes a significant shift (55% population showed shift) in fluorescence. Tetracycline does not cause membrane damage. The combination displays a similar shift to nalidixic acid-treated cells. Triton X-100 (0.01% v/v) acts as a positive control. Ten thousand total events were captured and are shown here. **b** Dose–response curve showing membrane damage caused by nalidixic acid. Membrane damage was assessed using SYTOX^TM^ Orange. Cells were incubated with increasing concentration of nalidixic acid and fluorescence was monitored over 1 h. Cells with no nalidixic acid acts as control (0% membrane damage). Effective concentration (EC_50_) for membrane damage was plotted using “dose response–inhibition, log [inhibitor] vs. normalized response parameter” in GraphPad Prism 7 software.

Surprised with our previous result of direct enhanced uptake of tetracycline by nalidixic acid, next we investigated the mode of action of this synergy using several assays for membrane integrity such as direct measurement of membrane damage and dissipation of proton motive force (PMF). To study whether tetracycline uptake induced by nalidixic acid is due to direct membrane disruption in *A. baumannii* AYE, we performed membrane permeability assay induced by nalidixic acid using a membrane-impermeable dye SYTOX™ Orange. SYTOX™ Orange can only enter the cells with a compromised membrane. Flow cytometry histograms of *A. baumannii* AYE cells treated with nalidixic acid displayed a shift to higher fluorescence intensities indicative of membrane disruption. Histogram of *A. baumannii* AYE cells treated with 1× MIC (512 mg/L) of nalidixic acid showed extensive membrane damage (55% cells displayed shift) (Fig. 3a). Tetracycline did not cause any fluorescence shift (≤1% cells displayed shift), indicating no role in membrane damage. The combination also displayed a similar shift (Fig. 3a), as was observed in *A. baumannii* AYE cells treated with nalidixic acid, indicating the possible role of membrane damage behind this synergy. Next, we also tested membrane damage assay with *E. coli* RPTU54, an MDR clinical strain of *E. coli* (nalidixic acid and tetracycline displayed synergy in this strain), and we found that nalidixic acid displayed a fluorescence shift (Supplementary Fig. 5), indicating that this membrane-damaging property of nalidixic acid at higher concentration might be conserved for all species. That is why, when we tested this on susceptible strains such as *A. baumannii* SDF and *E. coli* MG1655, nalidixic acid did not show any fluorescence shift, indicating no membrane damage at lower concentrations (Supplementary Fig. 5).

Next, we also performed a dose–response assay to check whether membrane damage by nalidixic acid is a concentration-dependent phenomenon. We used membrane-impermeable dye SYTOX™ Orange for this assay and, interestingly, we found that nalidixic acid caused dose-dependent membrane damage in *A. baumannii* AYE. The EC_50_ (effective concentration for 50% membrane damage) for membrane damage against *A. baumannii* AYE cells was 158.7 ± 33.2 µg/ml (for an OD readings at 600 nm (OD_600nm_) ~ 0.3 cells) (Fig. 3b). Overall, nalidixic acid displayed concentration-dependent membrane damage.

### Evaluating the role of PMF on nalidixic acid and tetracycline synergy

PMF is critical for bacteria survival and it may play a central role in showing synergy between two antibacterials as shown in the previous study^17^. PMF of a cell is composed of two components, i.e., transmembrane electric potential (ΔΨ) and proton gradient (ΔpH). The two components of the PMF, ΔΨ and ΔpH, are interdependent. Intentionally controlling ΔΨ and ΔpH through chemical combinations leads to synergy^17^. The PMF controls many constitutively expressed MDR efflux pumps, which provide bacteria with a first-line resistance against antibiotics^18^. To decipher the role of PMF-based efflux pumps in nalidixic acid and tetracycline synergy in *A. baumannii* AYE, a proton uncoupler, carbonyl cyanide m-chlorophenyl hydrazone (CCCP), was used. CCCP equilibrates both the transmembrane ΔpH and the transmembrane Δψ, and thus depletes PMF. The increasing concentration of CCCP (0, 5, 10, 15, 20, 25 µM) did not change the FICI of nalidixic acid and tetracycline combination, although the individual MICs changed (up to fourfold), indicating a possible role of efflux pumps in resistance of both antibiotics in *A. baumannii* AYE (Supplementary Fig. 6). In another set of experiments, direct measurement of PMF (ΔΨ) was performed using a fluorescent dye, Bis-(1,3-dibutylbarbituric acid)-trimethine oxonol [DiBAC_4_(3)]. The fluorescence of DiBAC_4_(3) changed in response to fluctuation in membrane potential. Fluorescence spectroscopy of DiBAC_4_(3) with respect to the addition of nalidixic acid, tetracycline, or its combination to *A. baumannii* AYE cells indicates that these two antibiotics are not involved directly in disrupting the membrane potential (Supplementary Fig. 7). In summary, these two experiments suggested that nalidixic acid and tetracycline synergy does not depend on PMF in *A. baumannii* AYE.

### Assessing the role of ROS in nalidixic acid and tetracycline synergy in *A. baumannii* AYE

Antibiotics are known to cause generation of reactive oxygen species (ROS) that affect many cellular targets^19,20^. Hence, nalidixic acid, tetracycline, and its combination were tested for the generation of ROS to check whether the synergy is ROS dependent or not. 2′,7′-Dichlorofluorescein diacetate is an oxidative sensitive dye; after intracellular de-esterification followed by oxidation, it turns to highly fluorescent 2′,7′-dichlorofluorescein. Addition of nalidixic acid (1× MIC, 512 mg/L), tetracycline (1× MIC, 64 mg/L), or both antibiotics in combination (nalidixic acid and tetracycline at 1× MIC of both) led to the generation of an equal amount of ROS (Supplementary Fig. 8). Hence, the synergy of nalidixic acid and tetracycline cannot be explained on the basis of enhanced ROS generation in *A. baumannii* AYE.

### Nalidixic acid and tetracycline combination shows inter-species and strain-specific cell length heterogeneity

**Fig. 4:**
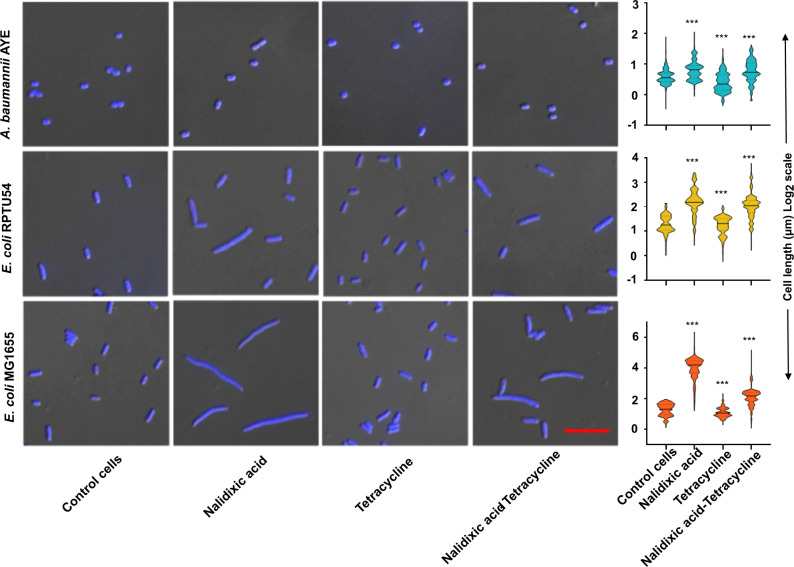
Cell length heterogeneity under nalidixic acid and tetracycline combination. Representative differential interface contrast (DIC) combined with fluorescence microscopy images of DAPI-stained (blue) *A. baumannii* AYE, *E. coli* MG1655, and *E. coli* RPTU54 cells growing in the absence of antibiotics (control cells) or in the presence of DNA synthesis inhibitors (nalidixic acid), translation inhibitor (tetracycline), or in a combination of both. Cell length displays the highest variability in the presence of DNA synthesis inhibitor but not in the presence of the translation inhibitor. All antibiotic concentrations were used at 0.75× MIC. Scale bar represents 10 µm. Morphometric analysis of cell length shown as bean plots. Major solid lines represent the medians, dotted lines represent 1/4^th^ and 3/4^th^ quartile. Minimum 500 cells were analyzed in each group. *P* < 0.001 for all three groups, one-way ANOVA followed by the Kruskal–Wallis post test was applied.

It is well documented that DNA damage in bacterial cells leads to cellular elongation^21^. The antagonism between bactericidal antibiotics such as nalidixic acid and bacteriostatic antibiotics such as tetracycline has been shown in previous studies^12,22^. In our study, *A. baumannii* AYE cells did not elongate or elongate marginally under nalidixic acid stress, whereas the nalidixic acid-susceptible population of *E. coli* MG1655 cells elongates several times of its original length (up to 50 times) (Fig. 4). *E. coli* RPTU54, a nalidixic acid-resistant strain, showed mixed phenotype, i.e., both normal cells and elongated cells were seen, but there was a remarkable difference in elongation between nalidixic acid-susceptible (greater elongation) and -resistant *E. coli* (lesser elongation) (Fig. 4). The morphometric analysis of individual cells (*n* ≈ 100 cells minimum) is shown as bean plots (Fig. 4). In summary, we observed a correlation between the type of antibiotic interaction and the level of antibiotic resistance.

### Role of mean total protein per cell and 16s rRNA gene expression in cell length heterogeneity caused by nalidixic acid and tetracycline combination

**Fig. 5:**
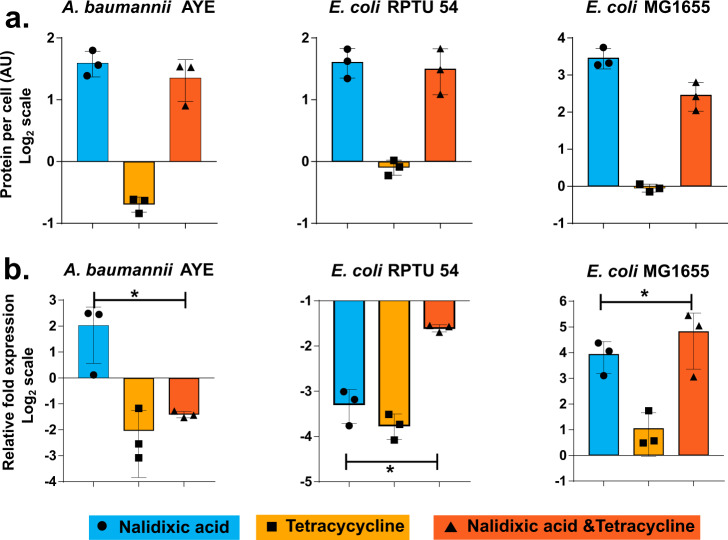
Role of total protein and 16s rRNA in nalidixic acid and tetracycline combination. **a** Mean total protein per cell in the presence of different antibiotics. Mean total protein per cell increases in the presence of nalidixic acid but not in the presence of tetracycline. All antibiotic concentrations were used at 0.75× MIC. Data represent mean and SD from three independent experiments. All values represent **p < 0.01; one-way ANOVA followed by the Kruskal–Wallis post test was applied. **b** Expression of 16s rRNA gene in the presence of various antibiotics normalized to no drug control. Relative expression of 16s rRNA gene increases in the presence of nalidixic acid but not in the presence of tetracycline. The combination of both antibiotics leads to an intermediate increase in expression. There is a remarkable difference in 16s rRNA gene expression between nalidixic acid-resistant (*A. baumannii* AYE and *E. coli* RPTU54) and -susceptible bacteria (*E. coli* MG1655). All antibiotic concentrations were used at 0.75× MIC. Data represent mean and SD from three independent experiments. All values represent **p < 0.01; one-way ANOVA followed by the Kruskal–Wallis post test was applied.

Mean total protein increases in the presence of DNA synthesis inhibitors and this increase is caused by excessive production of ribosomes in the *E. coli* MG1655 cells^22^. The ribosome is a core part of protein synthesis machinery in a bacterial cell and total protein in a cell correlates with the ribosome. Ribosomes are composed of three species of ribosomal RNA (rRNA) (16S, 23S, and 5S rRNA)^23^. Mean total protein of *E. coli* MG1655 susceptible to nalidixic acid increased up to approximately eight times when they were treated with nalidixic acid or with a combination of nalidixic acid and tetracycline, whereas mean total protein of *A. baumannii* AYE and *E. coli* RPTU54 resistant to nalidixic acid increased only up to approcimately two to three times as compared to untreated cells (Fig. 5a). Treatment with tetracycline reduced or did not change the mean total protein in *A. baumannii* AYE and *E. coli* RPTU54 cells.

Expression of 16s rRNA corroborated with our mean total protein data in these three bacterial cells. Expression of 16s rRNA of *E. coli* MG1655 susceptible to nalidixic acid increased by ~10- to 16-fold when they were treated with nalidixic acid, whereas expression of 16s rRNA of *A. baumannii* AYE and *E. coli* RPTU54 resistant to nalidixic acid increased only by ~2- to 4-fold, respectively (Fig. 5b). Overall, there is a remarkable difference in cell lengths between quinolone-susceptible *E. coli* MG1655 and quinolone-resistant *E. coli* RPTU54 or *A. baumannii* AYE. Cell length of quinolone-susceptible *E. coli* increased many folds after nalidixic acid treatment (either alone or in combination with tetracycline), whereas quinolone-resistant *E. coli* did not show such an increase. Our data showed, there is a correlation between cell length and mean total protein per cell that is controlled by 16s rRNA pool. It also showed a correlation between cell length and type of antibiotic interaction between nalidixic acid and tetracycline, i.e., synergy or no interaction. Bacteria that can increase cell length under stress (treatment with either nalidixic acid alone or in combination with tetracycline) showed no interaction type, whereas bacteria that cannot increase cell length showed synergistic drug interaction.

### Elucidating the role of efflux pumps in nalidixic acid and tetracycline synergy

**Fig. 6:**
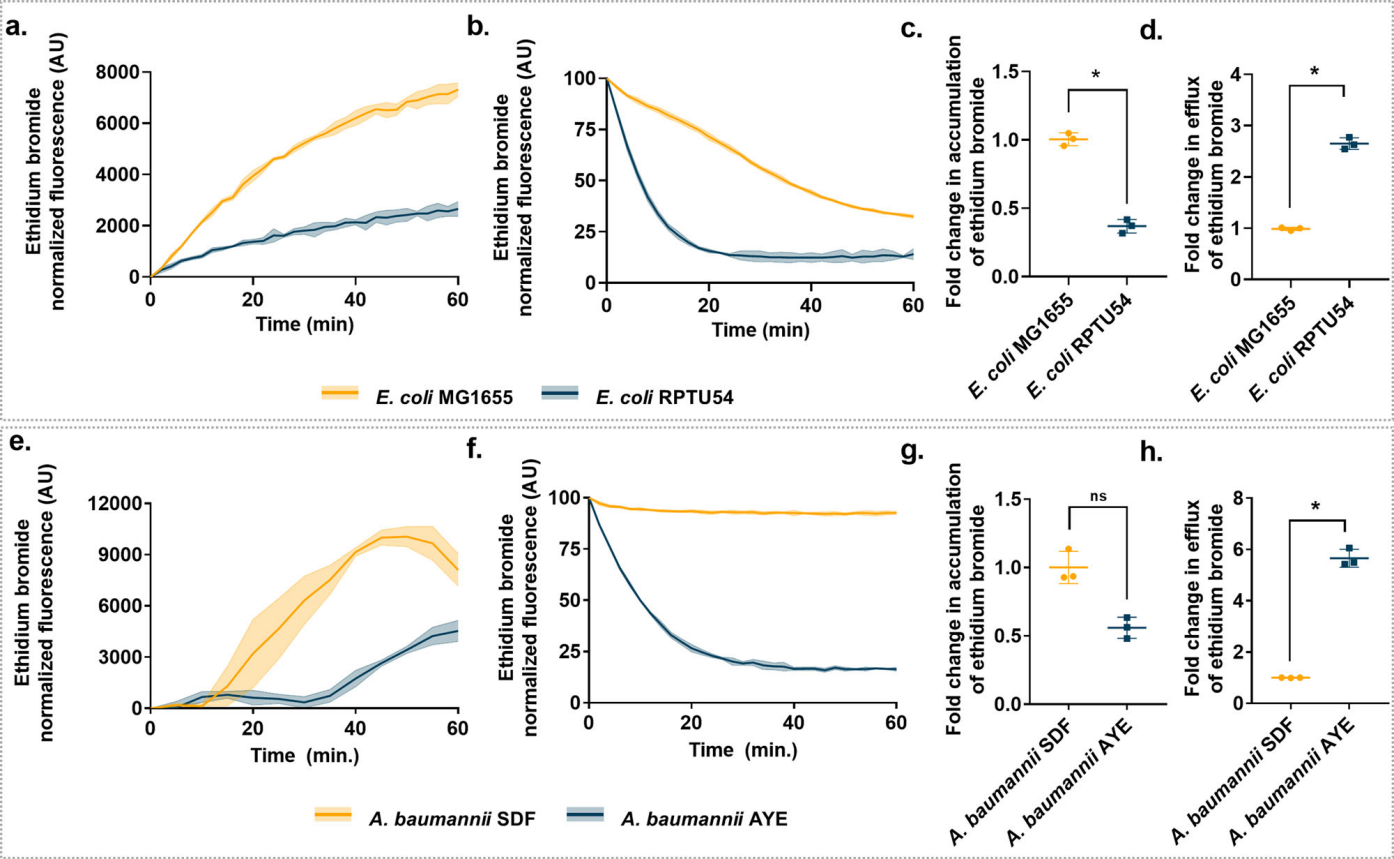
Difference of accumulation and efflux kinetics of ethidium bromide in quinolone-resistant vs. susceptible bacteria. **a** Accumulation kinetics, **b** efflux kinetics, **c** fold change in accumulation, and **d** fold change in efflux of ethidium bromide in *E. coli* MG1655 and *E. coli* RPTU54. **e** Accumulation kinetics, **f** efflux kinetics, **g** fold change in accumulation, and **h** fold change in efflux of ethidium bromide in *A. baumannii* SDF and *A. baumannii* AYE. Fold change was calculated from fluorescence reading obtained at the end of 1 h. Solid lines represent mean and shaded regions represents error bars (SD). * denotes a significant difference of *p* < 0.05, ns denotes not significant; Wilcoxon matched-pairs signed-rank test. Data represents mean and SD from three independent experiments.

Bacterial MDR efflux pumps are an important mechanism of antibiotic resistance. Cells with greater expression of an efflux pump will be less susceptible to various antimicrobials than its comparator with lower efflux pump expression. In our study, we compared the difference in activity level of efflux pumps at basal level between nalidixic acid-susceptible strains such as *A. baumannii* SDF and *E. coli* MG1655 with resistant strains such as *A. baumannii* AYE and *E. coli* RPTU54 using accumulation and efflux kinetics of ethidium bromide. *A. baumannii* AYE and *E. coli* RPTU54 cells effluxed out higher amount of ethidium bromide and accumulated reduced amount of ethidium bromide as compared to *A. baumannii* SDF and *E. coli* MG1655, respectively (~5.8 times higher efflux in *A. baumannii* AYE vs. *A. baumannii* SDF; similarly, ~2.8 times higher efflux in *E. coli* RPTU54 vs. *E. coli* MG1655, *p* < 0.05, Fig. 6). This assay demonstrated that resistant strains have enhanced activity of efflux pumps at basal level as compared to susceptible strains.

Next, we also performed efflux inhibition assay with two commonly used efflux substrates (indicator dyes), i.e., ethidium bromide and Hoechst 33342. Our efflux kinetics data showed that nalidixic acid caused a concentration-dependent efflux inhibition of both of these dyes (Fig. 2b and Supplementary Fig. 9). Efflux inhibition by nalidixic acid in *A. baumannii* AYE cells might be responsible for reduced efflux of tetracycline that leads to cell death. As resistant bacteria rely heavily on efflux pumps as a defense mechanism against multiple antibiotics, consequently, the effect of inhibition of these pumps would be high. Overall, enhanced uptake (as previously shown) and reduced efflux of tetracycline by nalidixic acid may partially explain the basis of synergy between these two antibiotics.

### In vivo efficacy of nalidixic acid and tetracycline combination in *C. elegans*

**Fig. 7:**
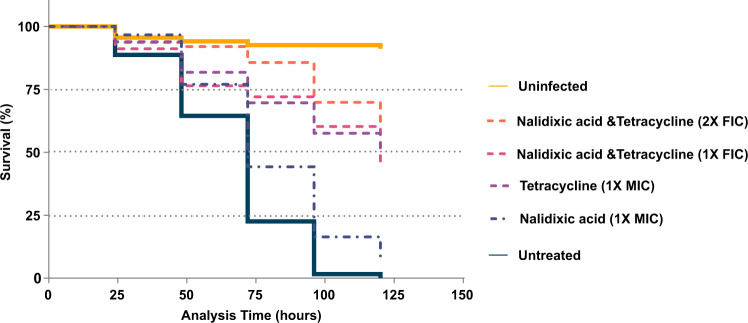
In vivo efficacy of nalidixic acid and tetracycline combination in *C. elegans*. Kaplan–Meier survival curve displaying synergy of nalidixic acid and tetracycline in the *C. elegans*–*A. baumannii* liquid infection model. Larvae (*n* ~ 60) were infected by *A. baumannii* AB5075-UW (a highly virulent MDR isolate) and treated with single antibiotics (nalidixic acid, tetracycline alone at 512 and 0.5 mg/L, respectively) or with a combination of antibiotics (1× FIC—nalidixic acid, tetracycline at 128 and 0.125 mg/L, respectively, or 2× FIC—nalidixic acid, tetracycline at 256 and 0.25 mg/L, respectively). One group was left untreated. The uninfected group was kept without bacterial infection and act as a negative control. The percentage of surviving larvae was monitored at indicated intervals after infection. Combination of nalidixic acid and tetracycline significantly prolonged larval survival; *p* < 0.001; log-rank (Mantel–Cox) test.

Finally, we evaluated the in vivo efficacy of nalidixic acid and tetracycline synergy using an established *C. elegans*–*A. baumannii* infection model. As previously reported, *A. baumannii* is pathogenic and lethal to the model host organism *C. elegans*, and this model was used to screen novel antibacterials^24^. The administration of nalidixic acid at 1× MIC (512 mg/L) did not protect the larvae against virulent *A. baumannii* AB5075-UW infection but tetracycline at 1× MIC (0.5 mg/L) protected 50% of the larvae (AB5075-UW is susceptible to tetracycline). In contrast, the combination of nalidixic acid and tetracycline prolonged their survival in a dose-dependent manner. More specifically, 60% and 50% of the larvae survived the lethal challenge for 120 h when supplemented with a combination of nalidixic acid and tetracycline at 2× FIC and 1× FIC (here FICI is 0.5), respectively (*p* < 0.001) (Fig. 7), demonstrating synergistic protection of larvae in vivo against highly virulent *A. baumannii* AB5075-UW infection.

## Discussion

Two antibiotics can interact quite differently depending on whether they are applied to drug-resistant mutants or drug-sensitive cells. In the current study, nalidixic acid and tetracycline was identified as a synergistic combination against *A. baumannii* AYE. Several studies have reported the antagonistic interactions between DNA synthesis inhibitors such as nalidixic acid and protein synthesis inhibitors such as tetracycline^4,11,12,22^. A recent high-throughput screening of pairwise drug interaction revealed the dichotomy about synergy and antagonism^10^. The study concluded that despite phylogenetic relatedness between *E. coli*, *S. enterica* serovar Typhimurium, and *P. aeruginosa*, only 30% of drug–drug interactions were common in these three species and rest of the 70% interactions were species-specific. In addition, 20% of them showed strain specificity^10^. Systematic exploration of drug interaction networks between 20 antibiotics representing the main modes of action in *E. coli* revealed that drug interactions occur frequently, but they are only partly predictable^12^. The results of our study also showed a similar pattern; most of the interactions were either indifferent or antagonistic. Moreover, the synergy between nalidixic acid and tetracycline, and not between ciprofloxacin and tetracycline highlights non-uniform class behavior, and it may be due to different chemical properties of the class members, and thus different dependencies on uptake and efflux systems^12^. Interactions between bactericidal and bacteriostatic antibiotics (which only inhibit growth) were long hypothesized to be predominantly antagonistic, as killing by bactericidal antibiotics often requires cell growth, which is prevented by bacteriostatic drugs. This view was validated in a recent study, which found that antagonistic interactions are significantly higher for bacteriostatic–bactericidal pairs among all pairwise combinations of 20 antibiotics^7,12^. However, one recent study reported synergy between bactericidal (enrofloxacin) and bacteriostatic (tigecycline) antibiotic against tetracycline-resistant *E. coli*, although the study did not focus on the mechanism of synergy^25^.

Our study has highlighted some key findings of nalidixic acid and tetracycline synergy against *A. baumannii* AYE. We found that the synergistic combination of nalidixic acid and tetracycline does not depend on PMF and ROS directly, although these two factors may contribute significantly for any given synergy^17,26^. Reduced PMF, in turn, impairs the function of multidrug efflux pumps that use PMF to export antibiotics from the cell, causing increased sensitivity to antibiotics^27^. In our study also, we observed a decrease in MIC of nalidixic acid and tetracycline in *A. baumannii* AYE upon treatment with increasing concentration of CCCP (a proton pump inhibitor) but there was no change in FICI. However, the membrane-damaging assay highlighted a potential role of nalidixic acid in membrane damage. Tetracycline uptake assay also pointed out the role of nalidixic acid in increasing intracellular concentration of tetracycline in *A. baumannii* AYE cells. This result correlates with the bioavailability model, which suggests that two drugs will be synergistic if one antibiotic helps another antibiotic’s availability in bacterial cells, either by increasing the second drug’s entry to the cell or by decreasing the second drug’s degradation or efflux^10,28^.

Morphometric analysis of cell length distribution under nalidixic acid and tetracycline stress revealed intra-species differences in antibiotic interaction. The combination of nalidixic acid and tetracycline acted antagonistically in *E. coli* MG1655 (susceptible to both antibiotics), whereas this combination acted synergistically in *E. coli* RPTU54 (resistant to both antibiotics). Our results corroborate a previous study showing that drug interactions may vary in resistant mutants^29^. Just a few mutations conferring resistance to one antibiotic can entirely change the cell’s response and interactions to other drugs. This raises the question as to what extent drug interactions are conserved in mutants and across microbial species. Although these principles need to be validated more broadly, they could play a key role in the future design of potent antibiotic combinations^5^. Antibiotic concentration is an important factor in controlling drug interactions. Multidrug efflux pumps control drug concentrations for bacterial cells. Our study highlighted the difference in the basal level activity of efflux pumps between drug-susceptible bacteria such as *A. baumannii* SDF and *E. coli* MG1655, and MDR *A. baumannii* AYE and *E. coli* RPTU54. Assessing in vitro drug interactions across a wide range of concentrations can guide in vivo studies, where variables such as absorption rates, elimination rates, and dosing regimens may lead to fluctuations in concentrations^30^. In our in vivo efficacy study also, combinations displayed synergistic activity by increasing the survival rates of *C. elegans* during infection.

Pharmacokinetics (PK) properties of nalidixic acid suggest that it can be dosed at very high concentrations. Nalidixic acid can be dosed at 4 g/day. Peak serum concentration of nalidixic acid is about 40 mg/L; however, peak urine concentration is about 150–200 mg/L after 1 g oral administration. In addition, active metabolism of nalidixic acid leads to generation of many metabolites. Unchanged nalidixic acid appears in the urine along with an active metabolite, hydroxynalidixic acid, which has antibacterial activity similar to that of nalidixic acid. Plasma concentration (100 mg/L) has been reported for hydroxynalidixic acid^31,32^. These concentrations are well above the concentrations required to kill pathogens such as *A. baumannii* AYE. The maximum serum drug concentrations (*C*_max_) of tetracycline after oral and intravenous dose is around 4 mg/L. However, we can achieve a high concentration of tetracycline in urine. Previous study has shown that administration of 1 g of tetracycline in a person with a normal renal function would excrete 60% for tetracycline in the urine. Assuming an output of urine of 1.5 l/day, maximal urinary levels would be 400 mg/L^33^. This concentration is quite high and is well above the concentration required to kill pathogens such as *A. baumannii* AYE. In addition, we may achieve high drug concentration if we are using antibiotic combination through nebulization for lung infection without systemic toxicity. Recently, we have shown that doxycycline along with polymyxin B could be used to treat acute pneumonia caused by MDR (doxycycline resistant) *P. aeruginosa* using a nebulizer in a mouse pneumonia model^34^. Overall, nalidixic acid and tetracycline combination may be used to treat urinary tract infections, lung infections, and skin infections caused by Gram-negative MDR pathogens such as *A. baumannii* and *E. coli*. As our in vitro cytotoxicity data suggest, the combination of nalidixic acid and tetracycline is non-toxic even at very high concentrations (IC_50_ ≥ 1024 + 256 mg/L, respectively (Supplementary Fig. 10).

However, there are some limitations of the current study. First, the current study did not look at PK properties of this combination, as PK properties play a central role in therapy; future studies may investigate the in vivo PK parameters of this synergy. Second, we found variation in type of drug interaction within a single antibiotic class, but our study has not investigated the reason for such variation in detail. It will be interesting to investigate the underlying molecular mechanisms, which may aid to identify novel synergistic partners in future. Third, this study typically focuses on one or a few bacterial representative strains of *A. baumannii* and *E. coli*; in future, we would like to validate our observations on other WHO priority pathogens.

Overall, our study highlighted the potential use of this combination for narrow-spectrum therapy, because it may prevent collateral damage to other beneficial pathogens^35–37^. Meanwhile, the identification of nalidixic acid and tetracycline combination encourages us to discover more candidates systematically as potential synergistic partners and antibiotic interaction studies represent a promising strategy to tackle the multiple drug-resistant bacterial pathogens.

## Methods

### Chemicals and biological materials

All antibiotics used in this study were obtained from Sigma Aldrich, St. Louis, MO, USA. *P. aeruginosa* ATCC 27853, *K. pneumoniae* ATCC 700603, *S. flexneri* ATCC 9199, *S. enterica* subsp*. enterica* serovar Choleraesuis ATCC 10708, *S. aureus* ATCC 29213, *A. baumannii* AYE (ATCC BAA-1710), *A. baumannii* SDF (ATCC BAA-1709), and *A. baumannii* ATCC 19606 were obtained from American Type Culture Collection, USA. *A. baumannii* AB5075-UW was obtained from Manoil Laboratory, UW Genome Sciences, University of Washington, USA. *M. smegmatis* and *E. coli* MG1655 were obtained from lab collection. The clinical isolates of *A. baumannii* (*n* = 16, obtained from Government Medical College and Hospital, Chandigarh, India) and *E. coli* (*n* = 3, obtained from Microbial Culture Repository Division of CIRD, Dr. B. Lal Clinical Laboratory Pvt Ltd, Jaipur, India) (listed in Supplementary Table 5) were minimally passaged and stored at −80 °C. All strains used in this study were routinely grown at 37 °C in Cation adjusted Mueller Hinton medium (HIMEDIA, India). *M. smegmatis* was grown in Mueller Hinton broth containing 0.1% Tween 80. Human breast cancer cell line (MCF-7) line was obtained from National Centre for Cell Science, Pune. Dulbecco’s modified Eagle medium (DMEM) and fetal bovine serum (FBS) were obtained from Sigma Aldrich, USA. *C. elegans fer-1* nematode and *E. coli* OP50 was obtained from Dr. Kavita Babu, Department of Biological Science, IISER Mohali, as a kind gift.

### Antibiotic susceptibility testing

MIC values of studied antimicrobials were determined with three biological replicates; each biological replicate contains three technical replicates. Assay was performed by broth microdilution method in 96-well polystyrene plate (Flat bottom sterile, Genaxy Scientific, India) with an initial inoculum of 10^6^ CFU/mL in Cation adjusted Mueller Hinton broth (CAMHB) according to CLSI guidelines^38^. Inoculum was prepared using actively growing mid-logarithmic phase cells. Plates were routinely incubated at 37 °C in a humidity saturated incubator (Kühner LT-X shaker, Adolf Kühner AG, Switzerland) to prevent edge effect. Growth was monitored by OD_600nm_ after 18 h of incubation using a Spectramax plus plate reader (Molecular Devices, USA).

### Screening of antibiotic–antibiotic combinations using checkerboard assay

In this screening, two quinolones—nalidixic acid and ciprofloxacin—were probed in *A. baumannii* AYE against antibiotics from different classes representing major classes of antibiotics such as ampicillin, fosfomycin, gentamicin, erythromycin, polymyxin B, tetracycline, and rifampicin. Two-dimensional microdilution checkerboard assays were performed in 96-well polystyrene plates (12 × 8 matrix, starting with 4× MIC), to unveil the antibiotic–antibiotic interaction effects of two selected antibiotic pairs. To evaluate the interaction of antibiotics, the FIC was calculated for each combination. FIC was calculated using the formula described below.

1 $${{\mathrm{FIC}}}_{\mathrm{A}}=\frac{{{\mathrm{MIC}}}_{\mathrm{A+B}}}{{{\mathrm{MIC}}}_{\mathrm{A}}}$$

2 $${{\mathrm{FIC}}}_{\mathrm{B}}=\frac{{{\mathrm{MIC}}}_{\mathrm{A+B}}}{{{\mathrm{MIC}}}_{\mathrm{B}}}$$

3 $${\mathrm{FICI}}={{{\mathrm{FIC}}}_{\mathrm{A}}+{\mathrm{FIC}}}_{\mathrm{B}}$$

FICI is defined as the sum of individual FICs. Synergy was defined as an FICI value of ≤0.5 and additivity or indifference was defined as an FICI value of ≥0.5 to <4, whereas antagonism was defined as an FICI value of ≥4^39^.

In this study, we aimed to identify the antibiotic combination that can rejuvenate activity of either nalidixic acid or ciprofloxacin against *A. baumannii* AYE.

### Time-kill kinetics

The time-kill experiments were performed in CAMHB (HIMEDIA, India) according to the previously described method with a slight modification^14^. An overnight culture of *A. baumannii* AYE raised from a single colony was diluted freshly 1 : 1000 in 5 ml CAMHB and incubated at 37 °C with aeration at 150 r.p.m. for 2 h. After 2 h, cells were diluted 1 : 100 in CAMHB containing 1× MIC of nalidixic acid alone (512 mg/L), 1× MIC of tetracycline alone (64 mg/L), 1× FIC (1/8th MIC of both), and 2× FIC (1/4th MIC of both) of the combination (here, for *A. baumannii* AYE, FICI is 0.25). Each tube received an initial bacterial inoculum of ~10^7^ CFU/mL. At indicated time intervals, 100 µl aliquots of cultures were removed and washed single time with 1× phosphate buffer saline (PBS), and were plated on CAMH agar plates after tenfold serial dilution in 1× PBS. Plates were incubated at 37 °C for 18 h. The time-kill curve was plotted using the mean colony count (Log_10_ CFU/mL) vs. time over 24 h.

### Membrane damage assay

Membrane damage assay was performed according to the previously described method with a slight modification^40^. Briefly, *A. baumannii* AYE cells were freshly grown till OD_600nm_ ~ 0.4 in CAMH broth and further diluted 1 : 1000 in CAMH broth with an addition of nalidixic acid, tetracycline alone (at 1× MIC corresponding 512 and 64 mg/L, respectively), or nalidixic acid and tetracycline combination (at 1× FIC corresponding 64 and 8 mg/L, respectively). *A. baumannii* SDF, *E. coli* RPTU54, and *E. coli* MG1655 cells were prepared similar to *A. baumannii* AYE; concentrations of antibiotic used for these bacteria are described in Supplementary Fig. 5. Triton X-100 (at 0.01% vol/vol) was added as a positive control. Cells were incubated for 4 h at 37 °C with shaking at 150 r.p.m. Cells were collected and washed twice in 1× PBS, having 0.4% glucose (wt./vol) at room temperature. SYTOX™ Orange (Invitrogen™, USA) was added to a final concentration of 5 µM and incubated for 10 min at room temperature in the dark. Cells (membrane damage) were analyzed using flow cytometer (BD FACSVerse™) with excitation at a wavelength of 547 nm and emission at a wavelength of 570 nm. For each sample, 10,000 events were recorded. Histograms (Half Offset) were analyzed and created using FlowJo™ Software for Windows v.10.0.4 (BD Biosciences, USA). SPHERO™ Rainbow Calibration Particles (BD Biosciences, USA) were used for instrument calibration.

For membrane damage dose–response assay, freshly grown *A. baumannii* AYE cells (OD_600nm_ ~ 0.4) were washed twice with 1× PBS, having 0.4% glucose (wt./vol), and subsequently resuspended in the same buffer at OD_600nm_ ~ 0.3. Next, SYTOX™ Orange (Invitrogen™, USA) was added to the cells at a final concentration of 5 µM at this stage. Then, nalidixic acid was added at different concentrations (0, 16, 32, 64, 128, 256, 512, and 1024 mg/L; 1× MIC of nalidixic acid represents 512 mg/L for this strain) in cell suspension and was immediately pipetted into the black polystyrene plates (Corning®, USA) at 100 µl/well. The fluorescence reading was monitored (at room temperature) using a Synergy^TM^ H1 Hybrid Multi-Mode fluorescence spectrophotometer (BioTek, USA) at an excitation wavelength of 545 nm and an emission wavelength of 570 nm for 60 min at every 5 min. The fluorescence readings were normalized as percentage with respect to 0 mg/L nalidixic acid fluorescence (i.e., 0% membrane damage) and were plotted against log_10_ concentration of nalidixic acid. Effective concentration (EC_50_) for membrane damage was plotted using “dose response–inhibition, variable slope parameter” in GraphPad Prism 7 software (GraphPad, La Jolla, California, USA).

### Tetracycline uptake assay

The tetracycline uptake assay was performed according to the previously described method^8^. The tetracycline uptake assay was monitored by fluorescence enhancement of tetracycline after cellular uptake. Cultures of *A. baumannii* AYE, *A. baumannii* SDF, *E. coli* MG1655, and *E. coli* RPTU54 were grown to OD_600nm_ ~ 0.6. Cells were pelleted down at 1200 × *g* for 10 min and resuspended in 10 mM HEPES buffer (pH 7.2) to a final OD_600nm_ ~ 0.5. Tetracycline was added to the cells at a final concentration of 128 mg/L at this stage. Then, nalidixic acid was added at different concentrations (for *A. baumannii* AYE 1×, 2×, 4×, and 8× MIC represents 512, 1024, 2048, and 4096 mg/L, respectively; concentrations of nalidixic acid used for *A. baumannii* SDF, *E. coli* MG1655, and *E. coli* RPTU54 is described in Supplementary Fig. 3) in cell suspension and finally pipetted into the black polystyrene plates (Corning®, USA) at 100 µl/well. The fluorescence reading was monitored (at room temperature) using a Synergy^TM^ H1 Hybrid Multi-Mode fluorescence spectrophotometer (BioTek, USA) at an excitation wavelength of 405 nm and an emission wavelength of 535 nm for 60 min at every 5 min. The fluorescence readings were normalized with respect to tetracycline fluorescence at time zero and plotted against time.

### Fluorescence microscopy and determination of protein per cell

Cultures of *A. baumannii* AYE, *E. coli* MG1655, and *E. coli* RPTU54 were grown till OD_600nm_ ~ 0.2 in fresh CAMH broth. Cells were treated with either nalidixic acid, tetracycline alone, or in combination for 3 h (each antibiotics and their combination were used at 0.75× of MIC, for *A. baumannii* AYE, 384 mg/L of nalidixic acid, or 48 mg/L of tetracycline was used either alone or in combination; for *E. coli* MG1655, 6 mg/L of nalidixic acid or 0.75 mg/L of tetracycline was used either alone or in combination; for *E. coli* RPTU54, 768 mg/L of nalidixic acid or 12 mg/L of tetracycline was used either alone or in combination). Cells were washed two times with 1× PBS at room temperature. Cells were stained with 4′,6-diamidino-2-phenylindole (DAPI) (Thermo Fisher Scientific, USA) at a concentration of 5 µg/mL for 5 min. Cells were mounted on agarose pad (1% wt./vol made in 1× PBS) and imaged with a fluorescence microscope equipped with DIC and DAPI filter set (Zeiss AxioScope.A1, Zeiss, Germany). Image processing was done using ZEN lite software. For morphometric analysis, cell lengths were measured manually using ImageJ software (NIH, http://imagej.nih.gov/ij/) for at least 500 cells (from 10 different field of view) in each condition.

To calculate protein per cell, 1 ml culture was collected after antibiotic treatment (each antibiotic or its combination were used at a concentration described above), washed in PBS, and lysed using BugBuster® Protein Extraction Reagent (Merck, Germany); total protein concentration was determined using Bradford reagent following the manufacturer’s instructions (HIMEDIA, India). Cell number was estimated by colony plate count on CAMH agar. Mean protein per cell was determined after dividing total protein by number of cells for each condition. The experiment was performed with three independent biological replicates.

### Synergy in the presence of uncoupling agent CCCP

PMF plays an important role in antibiotic susceptibility and drug interactions^17^. To check the role of PMF in nalidixic acid and tetracycline susceptibility, as well as their interaction against *A. baumannii* AYE, two-dimensional checkerboard assays (fixed 12 × 8 matrix containing nalidixic acid and tetracycline) were performed in 96-well plates with varying concentration (0, 5, 10, 15, 20, and 25 µM) of H^+^ uncoupler carbonyl cyanide m-chlorophenyl hydrazone (CCCP) (Sigma Aldrich, St. Louis, USA)^41^. CCCP causes the uncoupling of protons across the bacterial membranes and can change antibiotic susceptibility. The plate containing only antibiotics, i.e., nalidixic acid and tetracycline matrix without CCCP, acts as the control for this experiment.

### Spectrophotometric measurement of PMF disruption

Antibiotics can disturb PMF directly, which may be responsible for cellular lethality^17^. We also directly measured the role of nalidixic acid, tetracycline, or their combination on PMF in *A. baumannii* AYE. PMF was monitored using a potential-sensitive probe, DiBAC_4_(3) (Invitrogen™, USA). The fluorescence of DiBAC_4_(3) changes in response to fluctuation in membrane potential. *A. baumannii* AYE cells were grown till OD_600nm_ ~ 0.2 and washed twice in 1× PBS containing 0.4% glucose (wt./vol). Cells were pre-incubated with 10 µM of DiBAC_4_(3) for 10 min^42^. After 10 min, cells were treated with either nalidixic acid (at 512 mg/L), tetracycline (at 64 mg/L) alone, or in combination (nalidixic acid and tetracycline at 64 and 8 mg/L, respectively). Immediately after the addition of antibiotics, fluorescence reading was monitored (at 37 °C) using a Synergy^TM^ H1 Hybrid Multi-Mode fluorescence spectrophotometer (BioTek, USA) at an excitation wavelength of 490 nm and an emission wavelength of 516 nm for 60 min at every minute. Triton X-100 (0.01% vol/vol) acts as positive control for this experiment.

### Measurement of ROS using flow cytometry

A fluorescent probe—H2DCF-DA dye (Invitrogen™, USA)—was used to detect the amount of ROS generated upon antibiotic treatment. Briefly, *A. baumannii* AYE cells were grown till OD_600nm_ ~ 0.5, collected, and washed with 1× PBS containing 0.4% glucose (wt./vol). Cells were treated with either nalidixic acid (at 512 mg/L), tetracycline (at 64 mg/L) alone, or in combination (nalidixic acid and tetracycline at 64 and 8 mg/L, respectively) for 4 h. After treatment, cells were collected and centrifuged at 5000 r.p.m. at room temperature. The cells were washed twice with 1× PBS and resuspended in 1× PBS containing 5 μM H2DCF-DA dye^43^. Cells were incubated in the dark at room temperature for 10 min. After incubation, the cells were washed twice with 500 μl of 1× PBS to remove the excess dye and resuspended in 1× PBS for flow cytometry analysis (BD FACSVerse™). ROS was analyzed by flow cytometry with excitation at a wavelength of 488 nm and emission at a wavelength of 527–532 nm. For each sample, 10,000 events were recorded. SPHERO™ Rainbow Calibration Particles (BD Biosciences, USA) were used for instrument calibration. Histograms (Half Offset) were analyzed and created using FlowJo™ Software for Windows v.10.0.4 (BD Biosciences, USA).

### RNA isolation, cDNA synthesis, and quantitative RT-PCR

Quantitative reverse transcription PCR (RT-PCR) was performed according to the previously described method with modification^10^. Overnight cultures of *A. baumannii* AYE, *E. coli* MG1655, and *E. coli* RPTU54 were diluted 1 : 1000 into 5 ml CAMH broth and grown at 37 °C to OD_600nm_ ~ 0.3. Cells were treated with either nalidixic acid or tetracycline alone, or their combination at 0.75× MIC (for *A. baumannii* AYE, 384 mg/L of nalidixic acid or 48 mg/L of tetracycline was used either alone or in combination; for *E. coli* MG1655, 6 mg/L of nalidixic acid or 0.75 mg/L of tetracycline was used either alone or in combination; for *E. coli* RPTU54, 768 mg/L of nalidixic acid or 12 mg/L of tetracycline was used either alone or in combination) followed by 3 h incubation at 37 °C with shaking at 150 r.p.m. Cells were collected and RNA was extracted using RNAiso Plus (Takara Bio, Inc., Japan). cDNA was prepared for RT-qPCR using First Strand cDNA Synthesis Kit (Thermo Fisher Scientific, USA). Relative gene expression was estimated by RT-qPCR as previously described using PowerUp™ SYBR® Green Master Mix, following the manufacturer’s instructions (Applied Biosystems, USA)^44,45^. For *A. baumannii groEL*, *ftsZ*, *secA*, and *gmk* were used as housekeeping genes. For *E. coli groES*, *ftsZ*, *secA*, and *gmk* were used as housekeeping genes^46^. Relative expression of gene of interest (16s rRNA) was normalized with respect to all four housekeeping genes. Primer sequences used in this study are described in Supplementary Table 6. All experiments were conducted in at least three biological replicates.

### Measurement of accumulation and efflux kinetics by efflux pump

Ethidium bromide accumulation and efflux assays were carried out as previously described with minor modification^47^. Ethidium bromide is a common substrate for efflux pumps in bacteria. Cultures of *E. coli* MG1655 and *E. coli* RPTU54 were grown to an OD_600nm_ ~ 0.6, washed with PBS, and finally resuspended in PBS to an OD_600nm_ ~ 0.2. For ethidium bromide accumulation assay, cultures were loaded with 20 µg/ml of ethidium bromide and 0.4% glucose (wt./vol), and were immediately aliquoted into a black 96-well plate. For the ethidium bromide efflux assay, cultures were preloaded with 20 µg/ml of ethidium bromide without glucose and were incubated for 30 min at room temperature. Cells were washed two times with PBS and finally resuspended in PBS supplemented with 0.4% glucose (wt./vol) (to initiate efflux) to an OD_600nm_ ~ 0.2. Fluorescence was measured using a Synergy^TM^ H1 Hybrid Multi-Mode fluorescence spectrophotometer (BioTek, USA) at an excitation wavelength of 520 nm and an emission wavelength of 590 nm for 60 min at every 2 min.

For efflux inhibition assay, cells were preloaded with 20 µg/ml of ethidium bromide or 5 µg/ml Hoechst 33342 (Thermo Fisher Scientific, USA) without glucose and were incubated for 30 min at room temperature. Cells were washed and resuspended in PBS to an OD_600nm_ ~ 0.2. Then, nalidixic acid was added at different concentrations (0, 16, 32, 64, and 128 mg/L; 1× MIC of nalidixic acid represents 512 mg/L for this strain) in cell suspension and immediately pipetted into the black polystyrene plates (Corning®, USA) at 100 µl/well. CCCP (100 µM) was used as a positive control for this experiment. The fluorescence reading was monitored as described above. For Hoechst 33342 dye, an excitation wavelength of 360 nm and an emission wavelength of 485 nm was used. The fluorescence readings were normalized as percentage with respect to 0 mg/L nalidixic acid fluorescence and plotted against time.

### In vitro cytotoxicity of nalidixic acid and tetracycline combination

Cytotoxicity on human cell line (MCF-7) was performed by fluorescence-based resazurin (Sigma Aldrich, USA) assay as described earlier with minor modification^48,49^. Cells (1 × 10^5^) were incubated with nalidixic acid alone (32–2048 mg/L), tetracycline alone (8–512 mg/L), or nalidixic acid and tetracycline combination (starting with 32 mg/L of nalidixic acid and 8 mg/L of tetracycline till 2048 mg/L of nalidixic acid and 512 mg/L of tetracycline) in 96-well plates, and cultured in DMEM (Sigma Aldrich, USA) supplemented with 10% heat-inactivated FBS (Sigma Aldrich, USA) at 37 °C for 24 h, followed by resazurin tests. Cells without antibiotic acts as control (0% cytotoxicity) in this experiment.

### In vivo efficacy of nalidixic acid and tetracycline combination in *C. elegans* infection assay

*C. elegans* is a known pathogenesis model for various human pathogens such as *A. baumannii*^24^. *A. baumannii*–*C. elegans* infection assay was performed in a liquid medium as described previously, with slight modifications^24^. *C. elegans fer-1* was routinely maintained on nematode growth medium plates at 16 °C supplemented with *E. coli* OP50 as a food source. The *C. elegans fer-1* strain was used, because it becomes sterile at 25 °C. The infection assay was carried out in 96-well polystyrene plates in a final volume of 200 µL. Assay media consisted of 90% M9 buffer (Amresco, Inc., USA) supplemented with 2 mM MgSO_4_, 100 µM CaCl_2_, 10 µM FeCl_3_, and 10% CAMH broth along with nalidixic acid (512 mg/L), tetracycline (0.5 mg/L), or the combination (1× FIC—nalidixic acid and tetracycline at 128 and 0.125 mg/L, respectively; 2× FIC—nalidixic acid and tetracycline at 256 and 0.25 mg/L, respectively). For infection study, we used opaque colony of *A. baumannii* AB5075-UW (a highly virulent MDR strain) at 10^5^ CFU/mL (OD_600__nm_ ~ 0.01)^50^. Fifteen to 17 worms (L3 stage) were dispensed in each well manually, using an electronic 12-channel pipette (Xplorer® Plus, Eppendorf, Germany) at low dispensing speed to minimize physical injury to worms. One group of larvae was kept uninfected and served as controls for this experiment. Deaths were scored every 24 h during a total incubation period of 120 h using four biological replicates (each replicate contains a minimum of 15 *C. elegans*, *n* ≈ 60 total). Survival curves were plotted using the Kaplan–Meier method and differences in survival were calculated using the log-rank test in GraphPad Prism 7 software (GraphPad, La Jolla, California, USA). A *p*-value of ≤0.05 was considered to be statistically significant.

### Statistics and reproducibility

Results are expressed as the means ± SEM. Statistically significant differences were evaluated using appropriate tests as mentioned in the figure legends. In all instances, *p*-values ≤ 0.05 were considered as significant. Appropriate sample sizes are reported under specific methods and in figure legends. RT-PCR assay was performed with three independent biological replicates. The statistical analyses were performed using GraphPad Prism 7 software (GraphPad, La Jolla, California, USA).

### Reporting summary

Further information on research design is available in the Nature Research Reporting Summary linked to this article.

## Supplementary information

Supplementary Information

Description of Additional Supplementary Files

Supplementary Data 1

Supplementary Data 2

Reporting Summary

## Data Availability

The data that support the findings of this study are available from the corresponding author upon reasonable request. The Source data underlying Fig. 1c is provided as Supplementary Data 1. The Source data underlying Fig. 4 is provided as Supplementary Data 2.

## References

[1] WHO. *Global Priority List of Antibiotic-Resistant Bacteria to Guide Research, Discovery, and Development of New Antibiotics* (WHO, 2017).

[2] Wong D (2017). Clinical and pathophysiological overview of Acinetobacter infections: a century of challenges. Clin. Microbiol. Rev..

[3] Bush, K. in *Topics in Medicinal Chemistry - Antibacterials: Volume I* (eds Fisher, J. F., Mobashery, S. & Miller, M. J.) 69–88 (Springer, 2018).

[4] Bollenbach T (2015). Antimicrobial interactions: mechanisms and implications for drug discovery and resistance evolution. Curr. Opin. Microbiol..

[5] Chait R, Craney A, Kishony R (2007). Antibiotic interactions that select against resistance. Nature.

[6] Sullivan GJ, Delgado NN, Maharjan R, Cain AK (2020). How antibiotics work together: molecular mechanisms behind combination therapy. Curr. Opin. Microbiol..

[7] Jawetz E (1952). Antibiotic synergism and antagonism: review of experimental evidence. AMA Arch. Intern. Med..

[8] Ejim L (2011). Combinations of antibiotics and nonantibiotic drugs enhance antimicrobial efficacy. Nat. Chem. Biol..

[9] Eliopoulos GM, Eliopoulos CT (1988). Antibiotic combinations: should they be tested?. Clin. Microbiol. Rev..

[10] Brochado AR (2018). Species-specific activity of antibacterial drug combinations. Nature.

[11] Chandrasekaran S (2016). Chemogenomics and orthology-based design of antibiotic combination therapies. Mol. Syst. Biol..

[12] Ocampo PS (2014). Antagonism between bacteriostatic and bactericidal antibiotics is prevalent. Antimicrob. Agents Chemother..

[13] Dillon N (2019). Surprising synergy of dual translation inhibition vs. *Acinetobacter baumannii* and other multidrug-resistant bacterial pathogens. Ebiomedicine.

[14] Sopirala MM (2010). Synergy testing by Etest, microdilution checkerboard, and time-kill methods for pan-drug-resistant *Acinetobacter baumannii*. Antimicrob. Agents Chemother..

[15] Hutchings MI, Truman AW, Wilkinson B (2019). Antibiotics: past, present and future. Curr. Opin. Microbiol..

[16] Shang, D. et al. Synergistic antibacterial activity of designed Trp-containing antibacterial peptides in combination with antibiotics against multidrug-resistant *Staphylococcus epidermidis*. *Front. Microbiol*. **10**, 10.3389/fmicb.2019.02719 (2019). 10.3389/fmicb.2019.02719 PMC6886405 31824473

[17] Farha MA, Verschoor CP, Bowdish D, Brown ED (2013). Collapsing the proton motive force to identify synergistic combinations against *Staphylococcus aureus*. Chem. Biol..

[18] Griffith JM (2019). Experimental evolution of *Escherichia coli* K-12 in the presence of proton motive force (PMF) uncoupler carbonyl cyanide-chlorophenylhydrazone selects for mutations affecting PMF-driven drug efflux pumps. Appl. Environ. Microbiol..

[19] Kohanski MA, Dwyer DJ, Hayete B, Lawrence CA, Collins JJ (2007). A common mechanism of cellular death induced by bactericidal antibiotics. Cell.

[20] Dwyer, D. J., Kohanski, M. A., Hayete, B. & Collins, J. J. Gyrase inhibitors induce an oxidative damage cellular death pathway in *Escherichia coli*. *Mol. Syst. Biol*. **3**, 91 (2007). 10.1038/msb4100135 PMC1847949 17353933

[21] Nonejuie P, Burkart M, Pogliano K, Pogliano J (2013). Bacterial cytological profiling rapidly identifies the cellular pathways targeted by antibacterial molecules. Proc. Natl Acad. Sci. USA.

[22] Bollenbach T, Quan S, Chait R, Kishony R (2009). Nonoptimal microbial response to antibiotics underlies suppressive drug interactions. Cell.

[23] Maeda M, Shimada T, Ishihama A (2016). Strength and regulation of seven rRNA promoters in *Escherichia coli*. PLos ONE.

[24] Jayamani E (2015). Insect-derived cecropins display activity against *Acinetobacter baumannii* in a whole-animal high-throughput *Caenorhabditis elegans* model. Antimicrob. Agents Chemother..

[25] Liu Y (2020). Anti-HIV agent azidothymidine decreases Tet(X)-mediated bacterial resistance to tigecycline in *Escherichia coli*. Commun. Biol..

[26] Zou L (2018). Synergistic antibacterial activity of silver with antibiotics correlating with the upregulation of the ROS production. Sci. Rep..

[27] Lázár V (2013). Bacterial evolution of antibiotic hypersensitivity. Mol. Syst. Biol..

[28] Cokol, M. et al. Systematic exploration of synergistic drug pairs. *Mol. Syst. Biol*. **7**, 544 (2011). 10.1038/msb.2011.71 PMC3261710 22068327

[29] Wood KB, Wood KC, Nishida S, Cluzel P (2014). Uncovering scaling laws to infer multidrug response of resistant microbes and cancer cells. Cell Rep..

[30] Cottarel G, Wierzbowski J (2007). Combination drugs, an emerging option for antibacterial therapy. Trends Biotechnol..

[31] Vree TB, Wijnands WJ, Baars AM, Hekster YA (1988). Pharmacokinetics of nalidixic acid in man: hydroxylation and glucuronidation. Pharm. Weekbl. Sci..

[32] USFDA. *NegGram.pdf. NDA 14-214/S-058*, 3–12 (USFDA, 2008).

[33] Musher DM, Minuth JN, Thorsteinsson SB, Holmes T (1975). Effectiveness of achievable urinary concentrations of tetracyclines against “tetracycline-resistant” pathogenic bacteria. J. Infect. Dis..

[34] Gaurav A, Kothari A, Omar BJ, Pathania R (2020). Assessment of polymyxin B-doxycycline in combination against *Pseudomonas aeruginosa* in vitro and in a mouse model of acute pneumonia. Int. J. Antimicrob. Agents.

[35] Melander RJ, Zurawski DV, Melander C (2018). Narrow-spectrum antibacterial agents. Medchemcomm.

[36] Leekha S, Terrell CL, Edson RS (2011). General principles of antimicrobial therapy. Mayo Clin. Proc..

[37] Maier L (2018). Extensive impact of non-antibiotic drugs on human gut bacteria. Nature.

[38] CLSI. *Performance Standards for Antimicrobial Susceptibility Testing* (Clinical and Laboratory Standards Institute, 2018).

[39] Odds FC (2003). Synergy, antagonism, and what the chequerboard puts between them. J. Antimicrobial Chemother..

[40] Menzel LP (2017). Potent in vitro and in vivo antifungal activity of a small molecule host defense peptide mimic through a membrane-active mechanism. Sci. Rep..

[41] Krulwich TA, Sachs G, Padan E (2011). Molecular aspects of bacterial pH sensing and homeostasis. Nat. Rev. Microbiol..

[42] te Winkel JD, Gray DA, Seistrup KH, Hamoen LW, Strahl H (2016). Analysis of antimicrobial-triggered membrane depolarization using voltage sensitive dyes. Front. Cell Dev. Biol..

[43] Eruslanov E, Kusmartsev S (2010). Identification of ROS using oxidized DCFDA and flow-cytometry. Methods Mol. Biol..

[44] Pfaffl MW (2001). A new mathematical model for relative quantification in real-time RT-PCR. Nucleic Acids Res..

[45] Pfaffl, M. W. & Bustin, S. in *AZ of Quantitative PCR* 87–120 (International Univ. Line, 2004).

[46] Rocha DJP, Santos CS, Pacheco LGC (2015). Bacterial reference genes for gene expression studies by RT-qPCR: survey and analysis. Antonie Van Leeuwenhoek.

[47] Wang-Kan X (2017). Lack of AcrB efflux function confers loss of virulence on *Salmonella enterica* Serovar Typhimurium. Mbio.

[48] O’brien J, Wilson I, Orton T, Pognan F (2000). Investigation of the Alamar Blue (resazurin) fluorescent dye for the assessment of mammalian cell cytotoxicity. Eur. J. Biochem..

[49] Liu, Y. et al. Metformin restores tetracyclines susceptibility against multidrug resistant bacteria. *Adv. Sci*. **7**, 1902227 (2020). 10.1002/advs.201902227 PMC7312304 32596101

[50] Jacobs AC (2014). AB5075, a highly virulent isolate of *Acinetobacter baumannii* as a model strain for the evaluation of pathogenesis and antimicrobial treatments. Mbio.

