# Supplementary material for: Mechanistic insights into synergy between nalidixic acid and tetracycline against clinical isolates of Acinetobacter baumannii and Escherichia coli
Source: Commun Biol. 2021 May 10;4:542. doi: 10.1038/s42003-021-02074-5 (PMC8110569; doi:10.1038/s42003-021-02074-5)
Supplement: Supplementary file 1 — Supplementary Information [file 42003_2021_2074_MOESM1_ESM.pdf]

Supplementary Information

Mechanistic insights into synergy between nalidixic acid and tetracycline against clinical isolates of *Acinetobacter baumannii* and *Escherichia coli*

Amit Gaurav[a], Varsha Gupta[b], Sandeep K. Shrivastava[c], Ranjana Pathania[a]*

[a] Department of Biotechnology, Indian Institute of Technology Roorkee, Roorkee, Uttarakhand, India

[b] Department of Microbiology, Government Medical College and Hospital Chandigarh, Punjab, India

[c] Centre for Innovation, Research & Development, Dr. B. Lal Clinical Laboratory Pvt. Ltd. Jaipur, Rajasthan, India

* Address correspondence to: Ranjana Pathania, Tel: +91-1332-285324/+91-9761-305971; Fax: +91-1332-286151; E-mail: ranjana.pathania@bt.iitr.ac.in, ranjanapathania@gmail.com

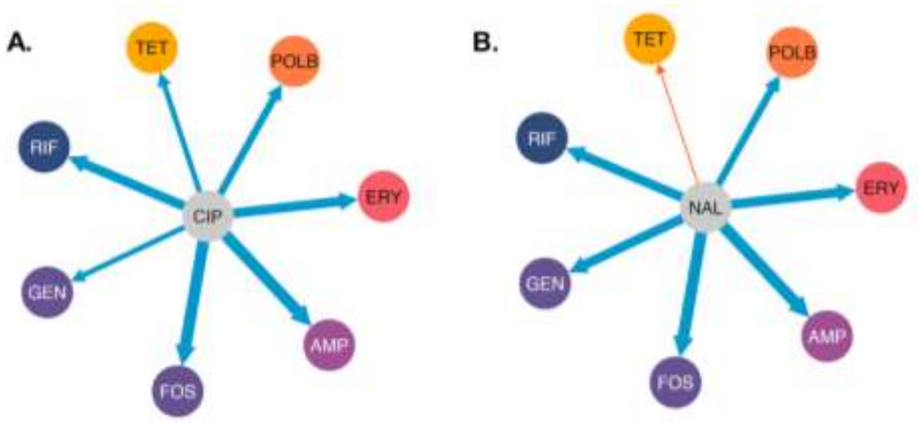

Edge	FICI	Interaction
	0.75	No Interaction
	1	No Interaction
	1.5	No Interaction
	2	No Interaction

Edge	FICI	Interaction
	0.25	Synergy
	1	No Interaction
	1.5	No Interaction
	2	No Interaction

Supplementary Figure. 1. Drug–drug interaction network of **(A.)** ciprofloxacin and **(B.)** nalidixic acid in *A. baumannii* AYE. Nodes represent different antibiotics, edges represent synergy (orange) or no interaction (blue), and thickness reflects the fractional inhibitory concentration index (FICI). Interaction network was created with Cytoscape version 3.8.0. TET, represents tetracycline; POLB, polymyxin B; ERY, erythromycin; AMP, ampicillin; FOS, Fosfomycin; GEN, gentamicin; RIF, rifampicin; CIP, ciprofloxacin and NAL, nalidixic acid.

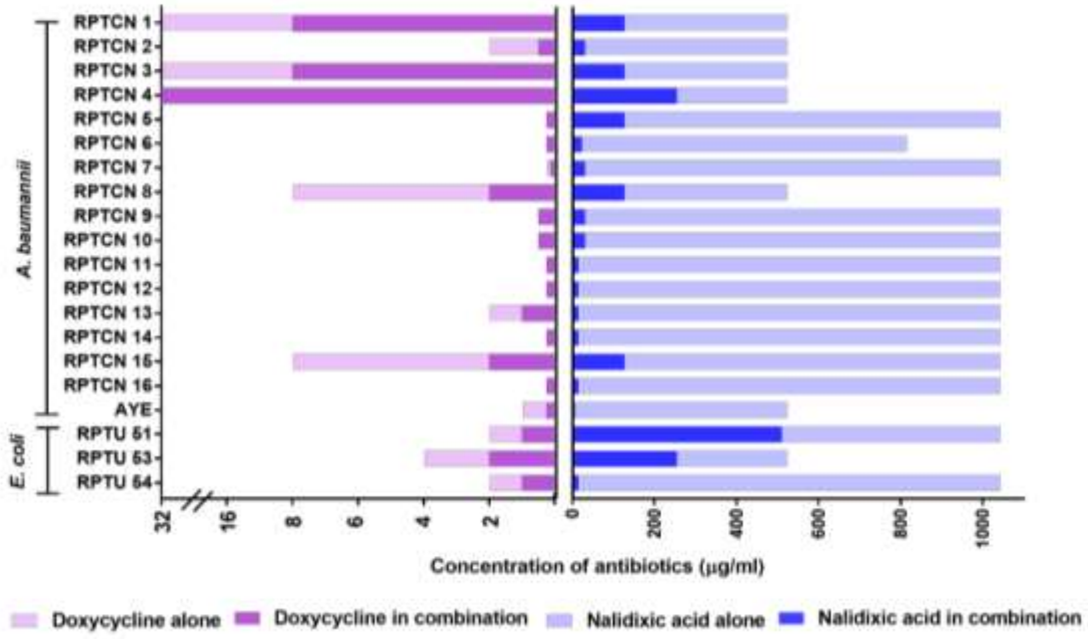

Supplementary Figure. 2. Concentration of doxycycline (dark purple) and nalidixic acid (dark blue) in combination, compared to doxycycline alone (light pink) and nalidixic acid alone (light blue) in clinical strains of *A. baumannii* and *E. coli*.

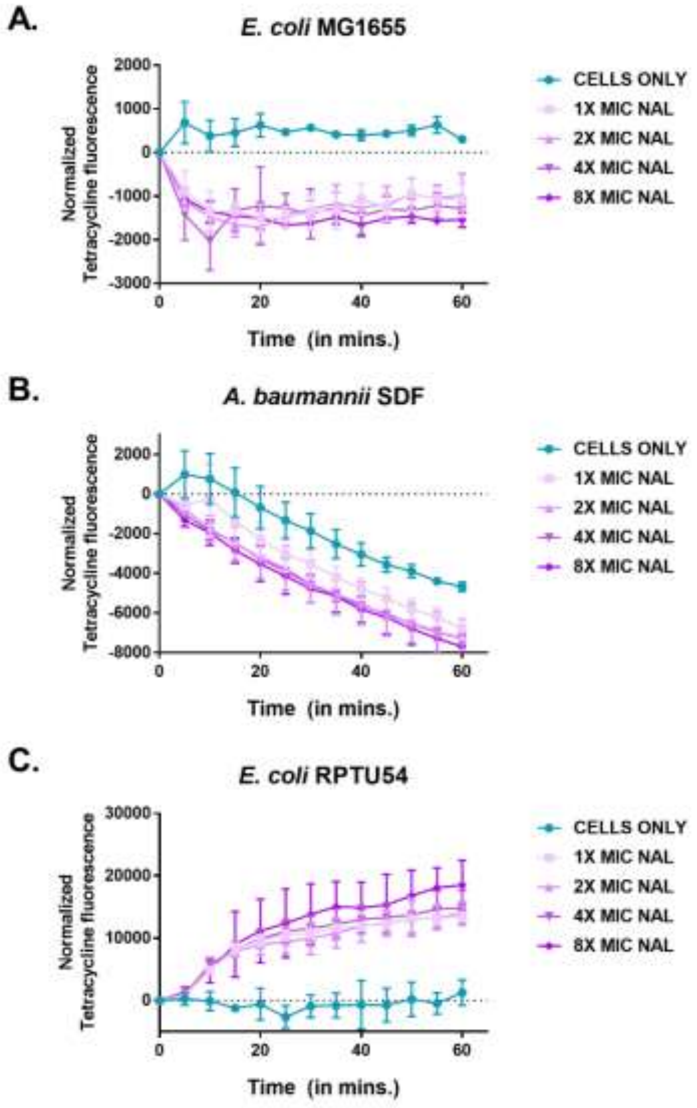

Supplementary Figure. 3. Tetracycline uptake assay. Nalidixic acid does not affect the uptake of tetracycline in *E. coli* MG1655 **(A.)** and *A. baumannii* SDF **(B.)** The concentration of tetracycline was kept constant at 128 mg/L. Here, 1X MIC of nalidixic acid represents 8 mg/L for *E. coli* MG1655 and 0.5 mg/L for *A. baumannii* SDF. However, nalidixic acid caused a concentration dependent increase in uptake of tetracycline in *E. coli* RPTU 54 **(C.)**. 1X MIC of nalidixic acid represents 1024 mg/L for *E. coli* RPTU 54. The increasing concentration of nalidixic acid was added as indicated in the subset above. Control cells represent bacterial cells without nalidixic acid treatment but with same amount of tetracycline. Tetracycline does not show intrinsic fluorescence

as “cells only,” i.e., cells without nalidixic acid treatment do not show an increase in fluorescence with time. Data are normalized with respect to tetracycline fluorescence at time zero. Data are represented as mean with standard deviation from three independent experiments.

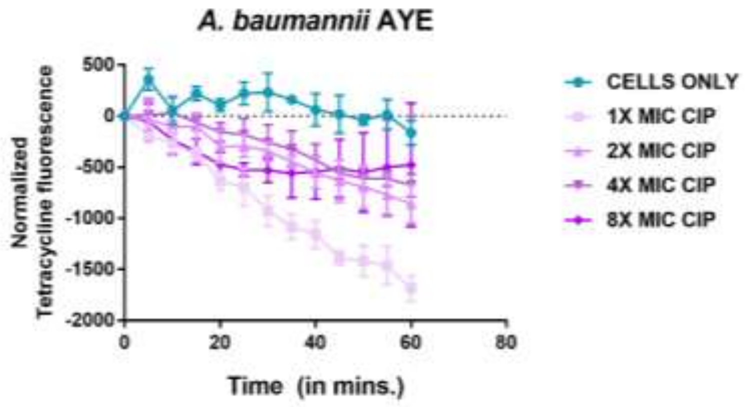

Supplementary Figure. 4. Tetracycline uptake assay. Ciprofloxacin does not affect the uptake of tetracycline in *A. baumannii* AYE. The concentration of tetracycline was kept constant at 128 mg/L. Here, 1X MIC of ciprofloxacin represents 512 mg/L for *A. baumannii* AYE.

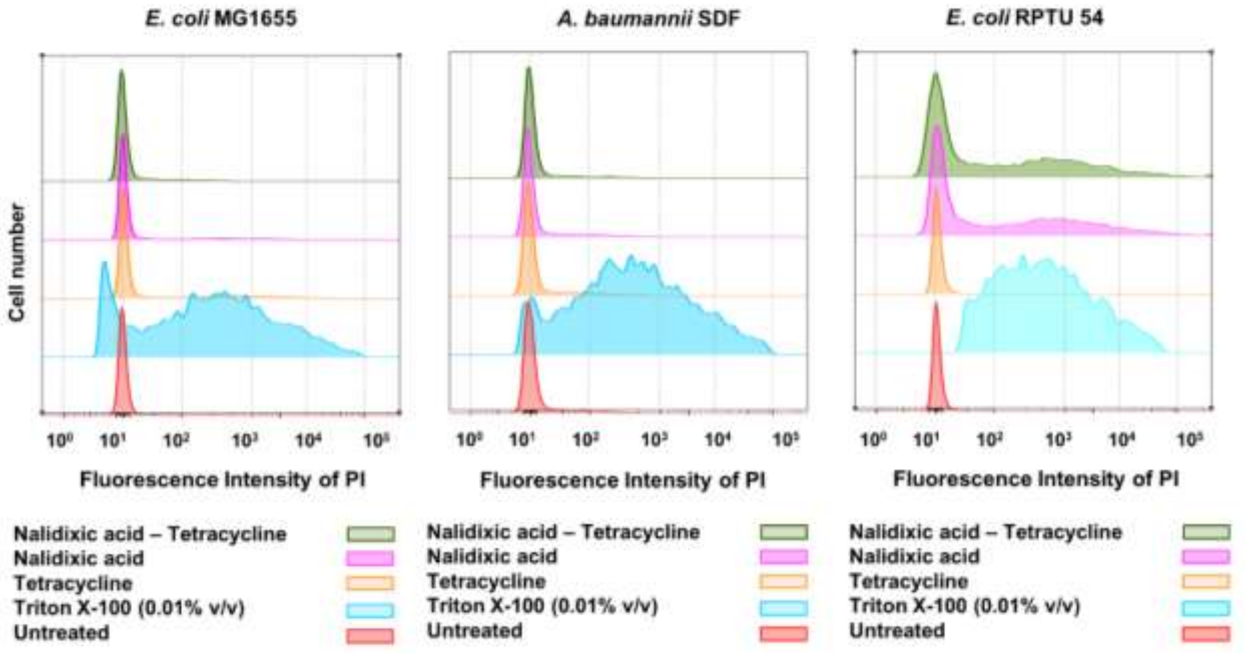

Supplementary Figure. 5. Flow cytometry analysis (Half Offset graphs) showing the role of nalidixic acid in membrane damage in *E. coli* MG1655, *A. baumannii* SDF and *E. coli* RPTU 54. Membrane damage was assessed using membrane-impermeable dye SYTOX^{TM} Orange. In *E. coli* MG1655, nalidixic acid treatment did not causes a significant shift in fluorescence. While in *E. coli* RPTU 54 nalidixic acid treatment caused a significant fluorescence shift (35% population showed shift). Tetracycline does not cause membrane damage. The combination displays a similar shift to nalidixic acid-treated cells. For *E. coli* MG1655, nalidixic acid, tetracycline alone was used at (8 & 1 mg/L respectively) or nalidixic acid and tetracycline combination (at 8 & 1 mg/L respectively). For *A. baumannii* SDF, nalidixic acid, tetracycline alone was used at (0.5 & 0.5 mg/L respectively) or nalidixic acid and tetracycline combination (at 0.5 & 0.5 mg/L respectively). For *E. coli* RPTU 54, nalidixic acid, tetracycline alone was used at (1024 & 16 mg/L respectively) or nalidixic acid and tetracycline combination (at 128 & 4 mg/L respectively). Triton X-100 (0.01% v/v) acts as a positive control. 10,000 total events were captured and are shown here.

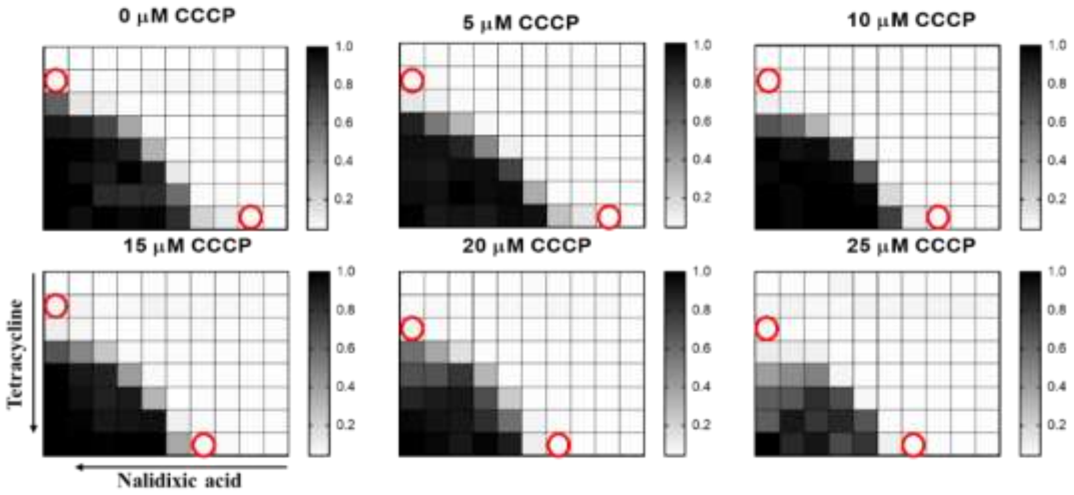

Supplementary Figure. 6. Effect of proton uncoupler carbonyl cyanide m-chlorophenyl hydrazone (CCCP) on nalidixic acid – tetracycline synergy in *A. baumannii* AYE. Nalidixic acid was diluted from right to left, and tetracycline from top to bottom. CCCP was used at the concentration described in the figure. The colour scale on the right side represents O.D.$_{600nm}$. CCCP is inhibitory for *A. baumannii* AYE cells at 100 µM. Increasing concentration of CCCP has decreased individual MIC of nalidixic acid and tetracycline in *A. baumannii* AYE, indicating the possible role of the efflux pump as one of the resistance mechanisms, but it has no effect on synergy.

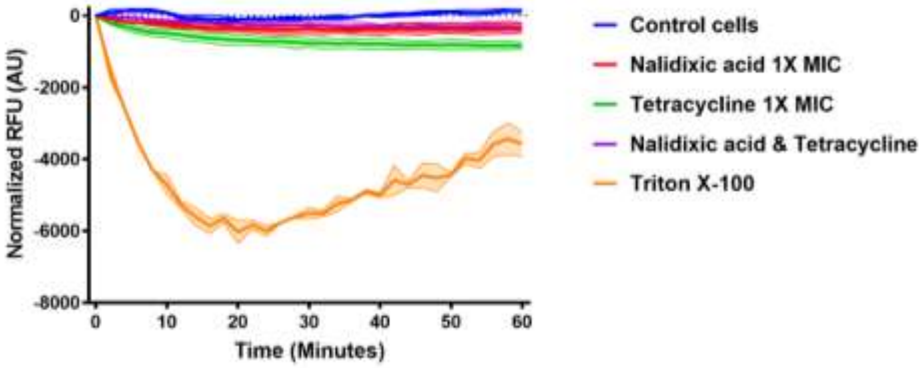

Supplementary Figure. 7. Effect of nalidixic acid – tetracycline combination on the membrane potential of *A. baumannii* AYE. Fluorescence intensity of $DiBAC_4(3)$ in *A. baumannii* AYE upon addition of different antibiotics and their combination listed in the subset above. As the inner membrane attains more negative (hyperpolarized), the anionic $DiBAC_4(3)$ leaves the cell, and its signal decreases. Conversely, as the inner leaflet of membrane attains more positive, more $DiBAC_4(3)$ enters the cells, and the $DiBAC_4(3)$ fluorescence signal increases. Triton X-100 (0.01% v/v) acts as positive control. Solid lines represent mean and shaded regions represents error bars (SEM). Data are normalized with respect to fluorescence intensity at time zero.

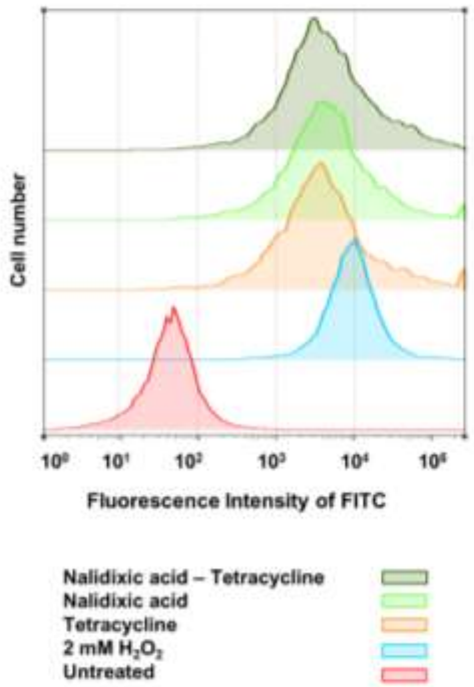

Supplementary Figure. 8. Flow cytometry analysis showing the role of different antibiotics in generating reactive oxygen species (ROS). Flow cytometry graphs (Half Offset) showing fluorescence shift of various antibiotic-treated *A. baumannii* AYE cells. Both nalidixic acid and tetracycline alone or in combination caused a significant generation of ROS as indicated by fluorescence shift (as ~ 65% population displayed relative shift) in spectra. H_2O_2 (2 mM) acts as positive control. 10,000 total events were captured and are shown here.

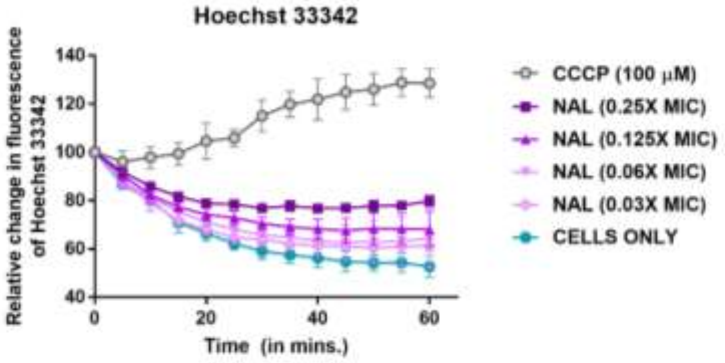

Supplementary Figure. 9. Hoechst 33342 efflux inhibition assay. Increasing concentration of nalidixic acid caused concentration dependent decrease in efflux of Hoechst 33342 (a common substrate for many efflux pumps). *A. baumannii* AYE cells without nalidixic acid "Cells only" caused maximum efflux of Hoechst 33342. Proton uncoupler carbonyl cyanide m-chlorophenyl hydrazone (CCCP) acts as efflux pump inhibitor (positive control) for this experiment.

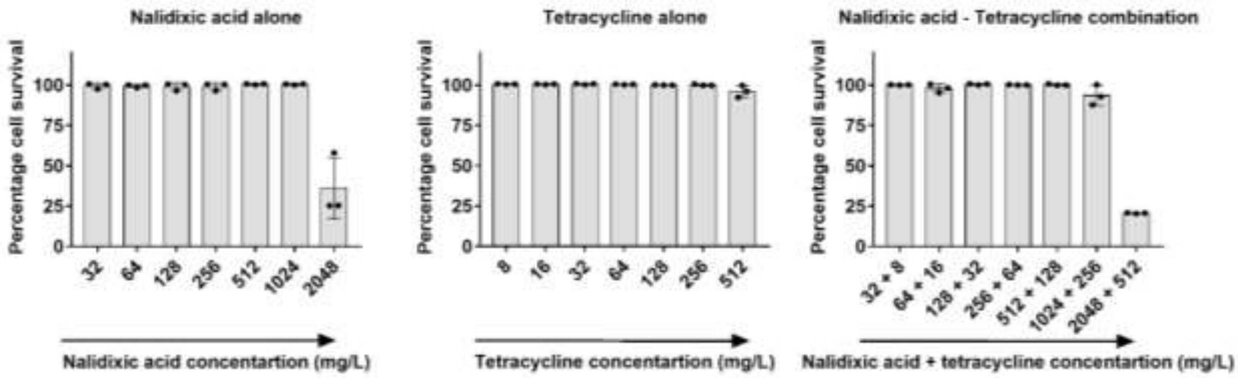

Supplementary Figure. 10. Cytotoxicity of nalidixic acid alone, tetracycline alone and combination of both in mammalian cell line (MCF-7). Cell viability was determined using resazurin assay after incubating antibiotics with cells for 24 hours. Here percentage cell survival is calculated with respect to no antibiotic treatment (100% cell survival).

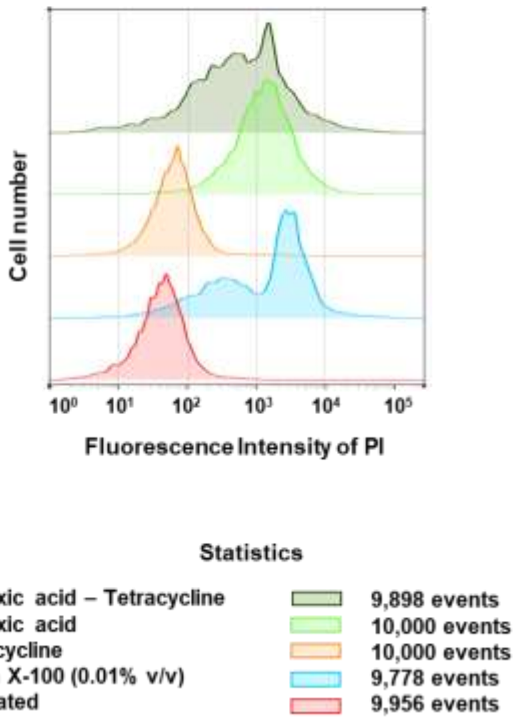

Supplementary Figure. 11. Gating strategy for flow cytometry analysis in figure 3a - showing the role of role of nalidixic acid in membrane damage. No gating was applied, only obvious cell debris were removed. More than 97% of cell population were included in the analysis.

Supplementary Table. 1. Table displaying minimum inhibitory concentration (MIC in mg/L), fractional inhibitory concentration (FIC), and fractional inhibitory concentration index (FICI) of various antibiotics with nalidixic acid (Nal) against *A. baumannii* AYE.

Antibiotics	MIC of antibiotic	MIC Nal	FIC Nal	FIC of antibiotic	FICI
Ampicillin	1024	512	1	1	2
Fosfomycin	64	512	1	1	2
Gentamicin	256	512	0.5	1	1.5
Erythromycin	256	512	1	0.5	1.5
Polymyxin B	0.5	512	0.5	0.5	1
Tetracycline	64	512	0.125	0.125	0.25
Rifampicin	4	512	1	0.5	1.5

Supplementary Table. 2. Table showing minimum inhibitory concentration (MIC in mg/L), fractional inhibitory concentration (FIC), and fractional inhibitory concentration index (FICI) of various antibiotics with ciprofloxacin (Cip) against *A. baumannii* AYE.

Antibiotics	MIC of antibiotic	MIC Cip	FIC Cip	FIC of antibiotic	FICI
Ampicillin	1024	512	1	1	2
Fosfomycin	64	512	1	1	2
Gentamicin	256	512	0.25	0.5	0.75
Erythromycin	256	512	1	0.5	1.5
Polymyxin B	0.5	512	0.5	0.5	1
Tetracycline	64	512	0.25	0.5	0.75
Rifampicin	4	512	1	0.5	1.5

Supplementary Table. 3. Table displaying minimum inhibitory concentration (MIC in mg/L), fractional inhibitory concentration (FIC), and fractional inhibitory concentration index (FICI) of nalidixic acid and tetracycline against various bacterial species.

Bacteria	Strain	MIC Nal	MIC Tet	FIC Nal	FIC Tet	FICI
A. baumannii	RPTCN 1	512	128	0.25	0.03125	0.28125
A. baumannii	RPTCN 2	512	64	0.125	0.0625	0.1875
A. baumannii	RPTCN 3	512	256	0.125	0.25	0.375
A. baumannii	RPTCN 4	512	256	0.25	0.0156	0.265
A. baumannii	RPTCN 5	1024	8	0.25	0.25	0.5
A. baumannii	RPTCN 6	800	2	0.25	0.5	0.75
A. baumannii	RPTCN 7	1024	32	0.25	0.25	0.5
A. baumannii	RPTCN 8	512	256	0.0625	0.125	0.1875
A. baumannii	RPTCN 9	1024	4	0.03125	0.5	0.53125
A. baumannii	RPTCN 10	1024	2	0.015625	0.5	0.515625
A. baumannii	RPTCN 11	1024	8	0.25	0.25	0.5
A. baumannii	RPTCN 12	1024	16	0.5	0.0625	0.5625
A. baumannii	RPTCN 13	1024	256	0.0625	0.125	0.1875
A. baumannii	RPTCN 14	1024	32	0.25	0.125	0.375
A. baumannii	RPTCN 15	1024	64	0.125	0.25	0.375
A. baumannii	RPTCN 16	1024	8	0.25	0.125	0.375
A. baumannii	AYE (ATCC BAA-1710)	512	64	0.125	0.125	0.25
A. baumannii	ATCC 19606	4	8	0.5	0.125	0.625
A. baumannii	SDF (ATCC BAA-1709)	0.5	0.5	1	1	2
A. baumannii	AB5075 UW	512	0.5	0.25	0.25	0.5
P. aeruginosa	ATCC 27853	256	128	0.5	0.03125	0.53125
K. pneomoniae	ATCC 700603	16	64	1	0.25	1.25
M. smegmatis	RPT45 (Lab collection)	16	1	1	1	2
S. flexneri	ATCC 9199	8	2	1	0.125	1.125
S. choleraesuis	ATCC 10708	16	32	1	1	2
S. aureus	ATCC 29213	32	4	1	0.5	1.5
E. coli	MG1655 (Lab collection)	8	1	2	1	3
E. coli	RPTU 51	1024	32	0.25	0.25	0.5
E. coli	RPTU 53	512	64	0.25	0.25	0.5
E. coli	RPTU 54	1024	16	0.125	0.125	0.25

Supplementary Table. 4. Table displaying minimum inhibitory concentration (MIC in mg/L), fractional inhibitory concentration (FIC), and fractional inhibitory concentration index (FICI) of nalidixic acid and doxycycline against various bacterial species.

Bacteria	Strain	MIC Nal	MIC Dox	FIC Nal	FIC Dox	FICI
A. baumannii	RPTCN 1	512	32	0.25	0.25	0.5
A. baumannii	RPTCN 2	512	2	0.0625	0.25	0.3125
A. baumannii	RPTCN 3	512	32	0.25	0.25	0.5
A. baumannii	RPTCN 4	512	32	0.5	0.5	1
A. baumannii	RPTCN 5	1024	0.25	0.125	1	1.125
A. baumannii	RPTCN 6	800	0.25	0.03125	1	1.03125
A. baumannii	RPTCN 7	1024	0.25	0.03125	0.5	0.53125
A. baumannii	RPTCN 8	512	8	0.25	0.25	0.5
A. baumannii	RPTCN 9	1024	0.5	0.03125	1	1.03125
A. baumannii	RPTCN 10	1024	0.5	0.03125	1	1.03125
A. baumannii	RPTCN 11	1024	0.25	0.015625	1	1.015625
A. baumannii	RPTCN 12	1024	0.25	0.015625	1	1.015625
A. baumannii	RPTCN 13	1024	2	0.015625	0.5	0.515625
A. baumannii	RPTCN 14	1024	0.25	0.015625	1	1.015625
A. baumannii	RPTCN 15	1024	8	0.125	0.25	0.375
A. baumannii	RPTCN 16	1024	0.25	0.015625	1	1.015625
A. baumannii	AYE (ATCC BAA-1710)	512	1	0.015625	0.25	0.265625
E. coli	RPTU 51	1024	2	0.5	0.5	1
E. coli	RPTU 53	512	4	0.5	0.5	1
E. coli	RPTU 54	1024	2	0.015625	0.5	0.515625

Supplementary Table. 5. Table showing minimum inhibitory concentration (MIC in mg/L) of various antibiotics against clinical strains of *A. baumannii* and *E. coli*.

**Bacteria**	**Strain**	**Piperacillin**	**Cefepime**	**Meropenem**	**Colistin**	**Polymyxin B**	**Gentamicin**	**Amikacin**	**Doxycycline**	**Minocycline**	**Tetracycline**	**Tigecycline**	**Nalidixic acid**	**Ciprofloxacin**	**Levofloxacin**	**Ofloxacin**	**Norfloxacin**	**Co-trimoxazole**
A. baumannii	RPTCN 1	>256	128	64	0.5	1	>256	>256	32	2	128	0.25	512	64	8	8	256	64
A. baumannii	RPTCN 2	>256	64	32	0.5	0.5	>256	>256	2	0.25	64	0.25	512	>256	32	32	256	32
A. baumannii	RPTCN 3	>256	>256	128	1	0.5	>256	>256	32	2	256	0.25	512	128	8	8	256	128
A. baumannii	RPTCN 4	>256	128	128	0.5	0.5	>256	>256	32	2	256	0.25	512	>256	32	32	256	128
A. baumannii	RPTCN 5	>256	>256	32	1	0.5	>256	>256	0.25	0.125	8	0.25	1024	128	16	16	256	64
A. baumannii	RPTCN 6	>256	>256	64	0.5	0.5	>256	>256	0.25	0.125	2	0.25	800	128	32	64	256	128
A. baumannii	RPTCN 7	>256	128	64	0.25	0.5	>256	>256	0.25	0.125	32	0.25	1024	256	16	32	256	128
A. baumannii	RPTCN 8	>256	32	32	0.5	0.5	>256	128	8	0.5	256	0.125	512	32	4	16	128	32
A. baumannii	RPTCN 9	>256	64	32	0.5	0.25	>256	>256	0.5	0.125	4	0.125	1024	64	4	32	256	16
A. baumannii	RPTCN 10	>256	64	32	0.5	0.5	>256	>256	0.5	0.125	2	0.125	1024	64	8	32	256	32
A. baumannii	RPTCN 11	>256	>256	32	0.5	0.5	>256	>256	0.25	0.125	8	0.25	1024	128	16	32	256	32
A. baumannii	RPTCN 12	>256	128	64	1	1	8	>256	0.25	0.125	16	0.25	1024	64	8	32	256	32
A. baumannii	RPTCN 13	>256	64	64	0.25	1	>256	>256	2	0.5	256	0.25	1024	256	16	64	256	128
A. baumannii	RPTCN 14	>256	64	32	0.25	0.5	16	>256	0.25	0.125	32	0.25	1024	128	8	32	256	32
A. baumannii	RPTCN 15	>256	>256	64	0.25	0.25	>256	>256	8	0.5	64	0.0625	1024	64	8	16	256	16
A. baumannii	RPTCN 16	>256	>256	16	0.25	0.25	128	64	0.25	0.125	8	0.25	1024	64	8	32	256	16
E. coli	RPTU 51	256	>256	128	0.5	0.25	16	128	2	0.5	32	0.125	1024	256	16	128	256	32
E. coli	RPTU 53	2	2	0.5	0.5	0.5	32	16	4	0.5	64	0.25	512	256	4	256	256	0.5
E. coli	RPTU 54	1	1	0.125	0.25	0.25	4	2	2	0.5	16	0.125	1024	>256	8	64	256	4

Supplementary Table. 6. Table showing qPCR primer sequences used in this study.

**Gene name**	**Forward Primer**	**Reverse Primer**
16s rRNA (*A. baumannii*)	5'- CAGCTCGTGTCGTGAGATGT - 3'	5'- CGTAAGGGCCATGATGACTT -3'
16s rRNA (*E. coli*)	5'- CAGCCACACTGGAACTGAGA - 3'	5'- GTTAGCCGGTGCTTCTTCTG - 3'
groEL (*A. baumannii*)	5' - GGTTACAACGCTGCAACTGG - 3'	CCACCCATACCGCCCATATC- 3'
groES (*E. coli*)	5' - CAATGGCCGTATCCTTGAAA - 3'	5' - AATTGCCAGAATGTCGCTTT - 3'
adeB (*A. baumannii*)	5' - ATGGCGAACAGTACGGAAGG- 3'	5' -TCTTGGCTGCCATTGCCATA- 3'
tet(A) (*A. baumannii*)	5' - GTTTGATGCGAACCGGCTTT- 3'	5' -GCTCGGTGGTATCTCTGCTC- 3'
omp33 (*A. baumannii*)	5' - ATCACGTGGGTTGCCATCTT- 3'	5' -TGTTGGTGCATCTTTTGCGG- 3'
ftsZ (*A. baumannii*)	5' - AAACGATGGCAACGGTCAAG- 3'	5' - ATGTGCTGAACGGCATTACC- 3'
ftsZ (*E. coli*)	5' - TGCATTTGCTTCCGACAACG- 3'	5' - ACGTTTGTCCATGCCGATAC- 3'
secA (*A. baumannii*)	5' - AACGTGGTTTGCACTATGCC- 3'	5' - TTGCGGGCGTAATTTTGGTG- 3'
secA (*E. coli*)	5' - TCATGCTGCAAACGCTTGAC- 3'	5' - TCGCTGCAAACATGGAGAAC- 3'
gmk (*A. baumannii*)	5' - AACAGCTTGCGTACTTGCTC- 3'	5' - TATGGCACTTCGCAAGCAAC- 3'
gmk (*E. coli*)	5' - ACCGCCGTCCAAAATTGAAC- 3'	5' - TGGCTCATTTCTGCAACAGC- 3'
