# Supplementary material for: Mechanistic insights into synergy between nalidixic acid and tetracycline against clinical isolates of Acinetobacter baumannii and Escherichia coli
Source: Commun Biol. 2021 May 10;4:542. doi: 10.1038/s42003-021-02074-5 (PMC8110569; doi:10.1038/s42003-021-02074-5)
Supplement: Supplementary file 2 — Description of Additional Supplementary Files [file 42003_2021_2074_MOESM2_ESM.pdf]

Description of Additional Supplementary Files

File Name: Supplementary Data 1

Description: Raw data for drug – drug interaction network

Sheet 1 – All Quinolones and Tetracyclines interaction (Figure 1.c)

Sheet 2 – Ciprofloxacin and Tetracyclines interaction (Supplementary Figure 1.a)

Sheet 3 – Nalidixic acid and Tetracyclines interaction (Supplementary Figure 1.b)

Sheet 4 – (Figure 1.b)

Sheet 5 – (Figure 2.a)

Sheet 6 – (Figure 2.b)

Sheet 7 – (Figure 2.c)

Sheet 8 – (Figure 3.b)

Sheet 9 – (Figure 5.a)

Sheet 10 – (Figure 5.b)

Sheet 11 – (Figure 6.a, 6.b, 6.e, 6.f)

Sheet 12 – (Figure 7)

File Name: Supplementary Data 2

Description: Individual bacterial cell length used for morphometric analysis.

Sheet 1 - *Acinetobacter baumannii* AYE

Sheet 2 – *E. coli* RPTU54

Sheet 3 - *E. coli* MG1655
