# Supplementary material for: Mechanistic insights into synergy between nalidixic acid and tetracycline against clinical isolates of Acinetobacter baumannii and Escherichia coli
Source: Commun Biol. 2021 May 10;4:542. doi: 10.1038/s42003-021-02074-5 (PMC8110569; doi:10.1038/s42003-021-02074-5)
Supplement: Supplementary file 5 — Reporting Summary [file 42003_2021_2074_MOESM5_ESM.pdf]

nature research

Corresponding author(s): Ranjana Pathania

Last updated by author(s): Mar 22, 2021

Reporting Summary

Nature Research wishes to improve the reproducibility of the work that we publish. This form provides structure for consistency and transparency in reporting. For further information on Nature Research policies, see our Editorial Policies and the Editorial Policy Checklist.

Statistics

For all statistical analyses, confirm that the following items are present in the figure legend, table legend, main text, or Methods section.

n/a	Confirmed	
[ ]	[x]	The exact sample size (*n*) for each experimental group/condition, given as a discrete number and unit of measurement
[ ]	[x]	A statement on whether measurements were taken from distinct samples or whether the same sample was measured repeatedly
[ ]	[x]	The statistical test(s) used AND whether they are one- or two-sided<br>*Only common tests should be described solely by name; describe more complex techniques in the Methods section.*
[x]	[ ]	A description of all covariates tested
[x]	[ ]	A description of any assumptions or corrections, such as tests of normality and adjustment for multiple comparisons
[ ]	[x]	A full description of the statistical parameters including central tendency (e.g. means) or other basic estimates (e.g. regression coefficient) AND variation (e.g. standard deviation) or associated estimates of uncertainty (e.g. confidence intervals)
[ ]	[x]	For null hypothesis testing, the test statistic (e.g. *F*, *t*, *r*) with confidence intervals, effect sizes, degrees of freedom and *P* value noted<br>*Give P values as exact values whenever suitable.*
[x]	[ ]	For Bayesian analysis, information on the choice of priors and Markov chain Monte Carlo settings
[x]	[ ]	For hierarchical and complex designs, identification of the appropriate level for tests and full reporting of outcomes
[x]	[ ]	Estimates of effect sizes (e.g. Cohen's *d*, Pearson's *r*), indicating how they were calculated

Our web collection on statistics for biologists contains articles on many of the points above.

Software and code

Policy information about availability of computer code

Data collection	BD FACSuite™ for FACS data collection, ZEN (Blue edition) for microscopy
Data analysis	FlowJo™ Software for Windows v.10.0.4 for FACS data analysis<br>GraphPad Prism 7 for data analysis<br>ZEN (Blue edition) for microscopy image analysis<br>ImageJ for microscopy image analysis<br>Cytoscape for network analysis

For manuscripts utilizing custom algorithms or software that are central to the research but not yet described in published literature, software must be made available to editors and reviewers. We strongly encourage code deposition in a community repository (e.g. GitHub). See the Nature Research guidelines for submitting code & software for further information.

Data

Policy information about availability of data

All manuscripts must include a data availability statement. This statement should provide the following information, where applicable:

- Accession codes, unique identifiers, or web links for publicly available datasets
- A list of figures that have associated raw data
- A description of any restrictions on data availability

All data generated or analysed during this study are included in this published article (and its supplementary information files). No restrictions on data availability.

Field-specific reporting

Please select the one below that is the best fit for your research. If you are not sure, read the appropriate sections before making your selection.

- [x] Life sciences
- [ ] Behavioural & social sciences
- [ ] Ecological, evolutionary & environmental sciences

For a reference copy of the document with all sections, see nature.com/documents/nr-reporting-summary-flat.pdf

Life sciences study design

All studies must disclose on these points even when the disclosure is negative.

Sample size	No sample size calculation was performed; sample size was determined based on previous studies. Sample sizes were chosen to be minimally n=3 (unless specified otherwise in the figure legend), the minimal to allow for statistical analysis and to ensure reproducibility. For C. elegans study n=15 (4 technical replicates; total n=60) were chosen. For microscopic analysis at least 500 cells were analyzed per condition.
Data exclusions	No data was excluded.
Replication	To verify reproducibility of experiments, experiments were performed using biological replicates. Experiments were performed using 3 biological replicates, unless specified otherwise in the corresponding figure legend.
Randomization	There was no need for sample randomization, we worked with samples of bacterial cells.
Blinding	There was no need for blinding.

Reporting for specific materials, systems and methods

We require information from authors about some types of materials, experimental systems and methods used in many studies. Here, indicate whether each material, system or method listed is relevant to your study. If you are not sure if a list item applies to your research, read the appropriate section before selecting a response.

Materials & experimental systems

n/a	Involved in the study	
x		Antibodies
	x	Eukaryotic cell lines
x		Palaeontology and archaeology
x		Animals and other organisms
x		Human research participants
x		Clinical data
x		Dual use research of concern

Methods

n/a	Involved in the study	
x		ChIP-seq
	x	Flow cytometry
x		MRI-based neuroimaging

Eukaryotic cell lines

Policy information about cell lines

Cell line source(s)	National Centre for Cell Science (NCCS), Pune
Authentication	Authenticated by NCCS, Pune
Mycoplasma contamination	Contamination free cell lines were supplied by NCCS, Pune
Commonly misidentified lines (See ICLAC register)	NA

Flow Cytometry

Plots

Confirm that:

☒ The axis labels state the marker and fluorochrome used (e.g. CD4-FITC).

☒ The axis scales are clearly visible. Include numbers along axes only for bottom left plot of group (a 'group' is an analysis of identical markers).

☒ All plots are contour plots with outliers or pseudocolor plots.

☒ A numerical value for number of cells or percentage (with statistics) is provided.

Methodology

Sample preparation	A fluorescent probe - H2DCF-DA dye (Invitrogen™, USA) was used to detect the amount of reactive oxygen species (ROS) generated upon antibiotic treatment. Briefly, A. baumannii AYE cells were grown till OD600 ~0.5, harvested, and washed with 1X PBS containing 0.4% glucose (wt/vol). Cells were treated with either Nalidixic acid, Tetracycline alone, or in combination for 4 hours. After treatment, cells were collected and centrifuged at 5,000rpm at room temperature. The cells were washed twice with 1X PBS and resuspended in 1X PBS containing 5 µM H2DCF-DA dye. Cells were incubated in the dark at room temperature for 10 minutes. After incubation, the cells were washed twice with 500µl of 1X PBS to remove the excess dye and resuspended in 1X PBS for FACS analysis (BD FACSVerse™). ROS was analyzed by flow cytometry with excitation at a wavelength of 488nm and emission at a wavelength of 527 to 532nm. For each sample, 10,000 events were recorded. SPHERO™ Rainbow Calibration Particles (BD Biosciences, USA) were used for instrument calibration. Histograms (Half Offset) were analyzed and created using FlowJo™ Software for Windows v.10.0.4 (BD Biosciences, USA).
Instrument	BD FACSVerse™
Software	BD FACSuite™ for FACS data collection, FlowJo™ v.10.0.4 for data analysis.
Cell population abundance	Populations were not sorted.
Gating strategy	For analysis of H2CDF-DA fluorescence: no gating was applied, only obvious debris were gated out (mean fluorescence was analysed).

☒ Tick this box to confirm that a figure exemplifying the gating strategy is provided in the Supplementary Information.
